# Artificial intelligence reveals environmental constraints on colour diversity in insects

**DOI:** 10.1038/s41467-019-12500-2

**Published:** 2019-10-07

**Authors:** Shipher Wu, Chun-Min Chang, Guan-Shuo Mai, Dustin R. Rubenstein, Chen-Ming Yang, Yu-Ting Huang, Hsu-Hong Lin, Li-Cheng Shih, Sheng-Wei Chen, Sheng-Feng Shen

**Affiliations:** 1 Biodiversity Research Center, Academia Sinica, Taipei, 11529 Taiwan; 2 Institute of Information Science, Academia Sinica, Taipei, 11529 Taiwan; 3 Department of Ecology, Evolution and Environmental Biology, Columbia University, New York, NY 10027 USA; 4 Center for Integrative Animal Behavior, Columbia University, New York, NY 10027 USA; 5 Taiwan Endemic Species Research Institute, Nantou, 552 Taiwan

**Keywords:** Biogeography, Evolutionary ecology, Macroecology, Entomology

## Abstract

Explaining colour variation among animals at broad geographic scales remains challenging. Here we demonstrate how deep learning—a form of artificial intelligence—can reveal subtle but robust patterns of colour feature variation along an ecological gradient, as well as help identify the underlying mechanisms generating this biogeographic pattern. Using over 20,000 images with precise GPS locality information belonging to nearly 2,000 moth species from Taiwan, our deep learning model generates a 2048-dimension feature vector that accurately predicts each species’ mean elevation based on colour and shape features. Using this multidimensional feature vector, we find that within-assemblage image feature variation is smaller in high elevation assemblages. Structural equation modeling suggests that this reduced image feature diversity is likely the result of colder environments selecting for darker colouration, which limits the colour diversity of assemblages at high elevations. Ultimately, with the help of deep learning, we will be able to explore the endless forms of natural morphological variation at unpreceded depths.

## Abstract (web summary)

Deep learning has the potential to identify ecological relationships between environment and complex phenotypes that are difficult to quantify. Here, the authors use deep learning to analyse associations among elevation, climate and phenotype across ~2000 moth species in Taiwan.

## Introduction

Since Wallace^1^ and the early fascination with the biology of colours, research on animal colouration has become increasingly interdisciplinary through the integration of studies examining the relationship between the colour of an organism and its social or ecological environment with those of the proximate physiological and genetic mechanisms that generate these colours^2,3^. Such integrative studies of animal colouration have largely been driven by advances in technologies, such as spectrophotometry, digital imaging, computational neuroscience and large-scale comparative analyses^2^. Yet, explaining colour variation among animals at broad geographic scales even using these tools remains challenging^4^ due to the inherent difficulties of extracting informative colour and shape pattern features objectively^5–7^, a problem also faced in computer vision studies^8^. With the rapid rise of computer vision research and application—namely, the development of deep convolutional neural networks (CNN), a form of artificial intelligence (AI) that can learn dense and often abstract image features from millions of training images^9^—we are poised for a new generation of ecological and evolutionary studies of animal colouration. The key breakthrough of the deep learning method^10^ is that instead of relying on humans to teach computers to quantify human-defined image features (e.g. spatial distributions of the colours), the computer can learn on its own in an objective way which features to optimally place in which level of the model to achieve a desired task, such as object and pattern recognition, classification or prediction^8^. To the best of our knowledge, no animal colouration study has yet employed the deep learning method, which can potentially complement and improve upon the recent progress on colour pattern quantification^11–17^.

Here we use deep learning to obtain the key image features that can predict the elevational distribution of moth species in order to explore both how and why subtle differences in colouration vary with temperature along an elevational gradient. Unlike traditional approaches that are prone to human biases and constraints in our visual system^8^, the CNN of the deep learning approach enables objective description and comparison of local and global image features, including colour composition or pattern (e.g. spots, edges and stripes, wing and body shapes or other unknown subtle image features), although we acknowledge that the technique used to generate digital images can also be a source of bias^12^. First, we employ a transfer learning method and adopt the residual network (ResNet^18^, which uses residual mapping to reduce training errors in deep neural networks; see Methods for details) with 50 layers pre-trained on ImageNet—a dataset of over 15 million labelled high-resolution images in more than 22,000 categories—as the convolutional part of our model. We then use a global average pooling layer to obtain a 2048-dimension feature vector. Finally, we design a regressor consisting of two fully connected layers to learn the mapping between the key image feature vector and elevational distributions (see Supplementary Fig. 1 for a visual representation of the complete model architecture). Images of the target animal group (moths) are randomly partitioned into training (80%) and validation (20%) datasets. We then train our model using an Adam optimizer, which adapts the learning rate for every parameter with consideration of the first and second moments of gradients during optimization. Since most moth species had multiple images in the validation dataset, we feed all of a species’ images into the model, and then average the predictions as the finalized result (see Methods for details).

Since our deep learning model generates a 2048-dimensional feature vector that accurately predicts the average elevation of moth species, we can be certain that this multidimensional feature vector represents image features that vary along the elevational gradient. Accordingly, we find that there is higher image feature variation in lower elevation assemblages than higher ones. We further use structural equation modelling to show that this reduced image feature diversity is the result of darker coloration in colder environments, which limits the diversity of image features in high elevation regions. Our study demonstrates that deep learning can help define image features more objectively than humans by revealing subtle differences of animals that are difficult to detect by subjective feature definition, such as elevational colour diversity patterns.

## Results

### Deep learning predicts elevational ranges

**Fig. 1:**
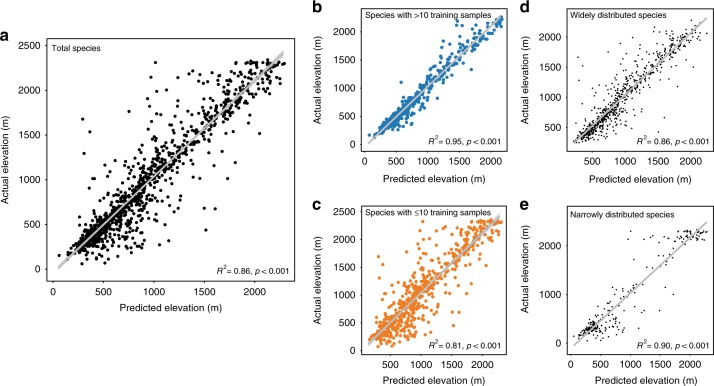
Comparison between the predicted and actual mean elevations of moth species. **a** There were 1951 and 1047 species in the complete and validation datasets, respectively. **b** For a subset of the data with >10 images in the training dataset, there were 500 and 480 species in the training and validation dataset, respectively. **c** For the subset of species with ≤10 images in the training dataset, there were 1451 and 567 species in the training and validation dataset, respectively. **d** For the subset of species with larger elevational distribution ranges (range size larger than the median size), there were 976 and 745 species in the training and validation dataset, respectively. **e** For the subset of species with smaller elevational distribution ranges (range size smaller than the median size), there were 975 and 302 species in the training and validation dataset, respectively. The results of a GLMM controlling for phylogenetic effects are shown at the bottom of each figure. Source data are provided as a Source Data file

**Fig. 2:**
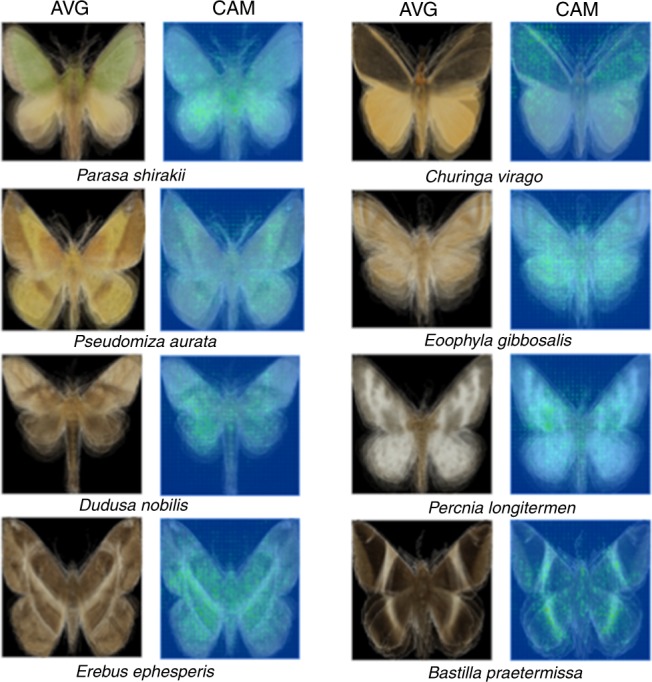
Class activation mapping generated localized discriminative visual features of arbitrarily selected images of eight species of moths. CAM represents class activation mapping, which is a method of localizing the discriminative image regions^59^. AVG shows the mean RGB of each pixel from images of all individuals of a species

To investigate how colour traits vary with the elevational distribution of moths, we first established the relationship between moth images and each species’ mean elevation. Our final deep learning model produced highly accurate predictions of a species’ mean elevation based only on moth images (Fig. 1a, GLM, *R*^2^ = 0.86, *p* < 0.001). The results remained robust after controlling for phylogenetic history by including family and genus as random effects in our model (generalized linear mixed-effect model (GLMM), *X*^2^ = 6163.6, *R*^2^ = 0.86, *p* < 0.001, *n* = 1047). We also tested for a sample size effect during training by separating moth species into those with large (>10 images) and small sample sizes (≤10 images). Our model generated accurate predictions about the mean elevation for those species with either large (Fig. 1b, GLMM, *X*^2^ = 9436.7, *R*^2^ = 0.95, *p* < 0.001) or small sample sizes (Fig. 1c, GLMM, *X*^2^ *=* 2133.9, *R*^2^ = 0.81, *p* < 0.001) as well as for widely distributed (Fig. 1d, GLMM, *X*^2^ = 4240.7, *R*^2^ = 0.86, *p* < 0.001) or narrowly distributed species (Fig. 1e, GLMM, *X*^2^ = 2114.9, *R*^2^ = 0.90, *p* < 0.001; see also Supplementary Figs. 2, 3 for variations of predicted elevations of individual images within a species without averaging the predicted elevations of individual images; GLMM, *X*^2^ = 12163.0, *R*^2^ = 0.82, *p* < 0.001) or excluding species that only appeared in one location (GLMM, *X*^2^ = 6543.2, *R*^2^ = 0.88, *p* < 0.001, Supplementary Fig. 4). Together, these results suggest that the 2048-dimension feature vector of our final model, which represents the colour traits of a species, accurately identified key moth image features that corresponded to a speciesʼ mean elevation (see Fig. 2 and Supplementary Fig. 5 for samples of image features identified by the deep learning model and our GitHub repository, https://github.com/twcmchang/colorful-moth, for the full dataset of image features). To further understand the image features used by the deep learning model, we retrained the models with greyscale and silhouette images to distinguish shape and greyscale patterns from colour-related image features. We found that the elevation predictions based on the greyscale and silhouette images were both substantially lower than models based on the colour images (greyscale images: Supplementary Fig. 6, GLMM, *X*^2^ = 559.8, *R*^2^ = 0.36, *p* < 0.001; silhouette images: Supplementary Fig. 7, GLMM, *X*^2^ = 563.3, *R*^2^ = 0.38, *p* < 0.001, compared with *R*^2^ = 0.86 based on the colour images). These results further suggest that colour features (e.g. colour composition and patterns) contribute more than half of the explained variance, and that both colour and shape information (and their interaction, e.g. the line, shape and form of colours) are important for predicting the mean elevation of a species.

### Colour diversity varies with elevation

**Fig. 3:**
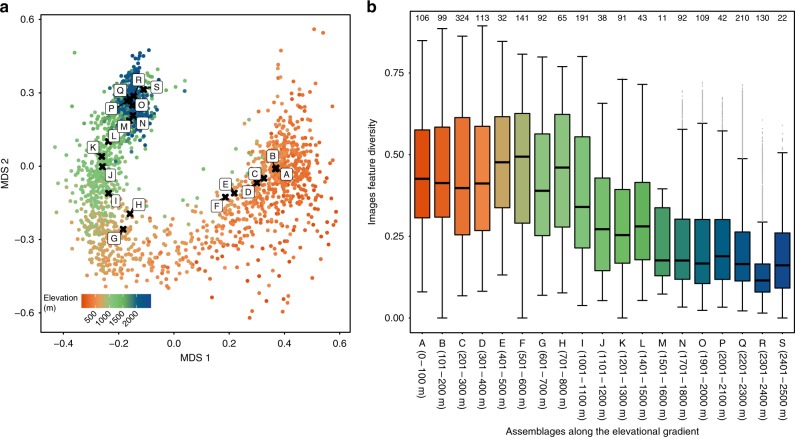
Multidimensional scaling (MDS) visualization of image features and within-assemblage image feature diversity of moths. **a** An MDS visualization of the 2048-dimension feature vector of species in relation to the moths’ elevational distribution. **b** Species assemblages along the elevational gradient were determined by their mean elevation. The legend of 0–100 m represents the assemblage of species mean elevations range from 0 to 100 m above sea level and so on, until 2400 to 2500 m. Since some intervals have no collection event, we acquired 19 species assemblages in total. Within-assemblage image feature diversity was defined as the cosine distance of their 2048-dimension feature vectors between any two species within the same assemblage. Warmer colours correspond to lower elevations, and cooler colours to higher elevations. In box-and-whiskers diagrams, boxes indicate median and upper and lower quartile and whiskers indicate range of data. Source data are provided as a Source Data file

To explore how moth colouration varies with temperature along the elevational gradient, we selected one specimen image for each species whose sampled elevation was closest to that species’ mean elevation as its representative image. By feeding a representative image into the trained ResNet model, we extracted the 2048-dimension output at the ResNet’s global average pooling layer as the species feature vector. We then quantified the trait distance between any two species as the cosine distance of their 2048-dimension feature vectors. Next, we projected the extracted 2048-dimension features into a two-dimensional map via multidimensional scaling (MDS) for visualization purposes (Fig. 3a). We found that a species’ distribution in two-dimensional trait space was largely based on its elevational distribution, which suggested that moth image features varied along the elevational gradient. We then conducted an assemblage-level analysis by grouping species according to their elevational distribution such that there was one assemblage per hundred metres (Fig. 3b). Each assemblage contained 11 to 324 species. We found that image feature diversity at the assemblage level—defined as the cosine distance of the 2048-dimensional feature vectors among species in an assemblage—was higher at low elevations (Fig. 3b, GLM, *F*_1,17_ = 122.2, *R*^2^ = 0.88, *p* < 0.001). To estimate the potential effect of different sample sizes among assemblages, we constructed 95% confidence intervals of the regression coefficients by bootstrapping with 5000 repeats^19^. In each repeat, we randomly resampled pairwise cosine distance from each assemblage to the size of assemblage M (with minimum number of species 11; therefore, 55 samples of pairwise cosine distance) with replacement. The estimations were close to our original result (GLM, intercept = [0.47, 0.50], elevation = [−0.00014, −0.00012], *R*^2^ = [0.78, 0.90], *F*_1,17_ = [52.5, 148.4], *p* < 0.001). To control for the potential effect of an elevational trend in β-diversity (Supplementary Fig. 8), we used the same resampling method described above in which we bootstrapped samples from four randomly selected families in each assemblage with 1000 repeats. The result remained qualitatively similar (GLM, intercept = [0.40, 0.46], elevation = [−0.00016, −0.00012], *R*^2^ = [0.71, 0.91], *F*_1,17_ = [30.7, 145.22], *p* < 0.001). To understand what information contributes more in explaining the elevational trend of image features, we used only greyscale images for the same analysis. We found that there was no significant elevational trend of image feature diversity in greyscale using the same resampling procedure (GLM, intercept = [0.26, 0.30], elevation = [−0.000048, −0.000020], *R*^2^ = [0.08, 0.57], *F*_1,17_ = [0, 23.07], *p* = [0, 0.12]), suggesting that colour-related image features are indeed the main features varying with the change of elevation in the 2048-dimensional feature vector. Finally, we grouped species with different elevational intervals and the trend remained the same (Supplementary Fig. 9a, GLM, *F*_1,24_ = 95.9, *R*^2^ = 0.80, *p* < 0.001; Supplementary Fig. 9b, GLM, *F*_1,10_ = 106.1, *R*^2^ = 0.91, *p* < 0.001).

### Biotic and abiotic factors drive image feature distributions

To determine how biotic and abiotic factors drive the trends in image feature distributions among assemblages observed along the elevational gradient, we performed a variance partitioning analysis to determine the percentage of variance of the image features that is attributed to differences across nested scales (i.e. population, assemblage and regional pool)^20,21^. A lower ratio of community/assemblage-wide trait variance to total variance in the regional pool suggests stronger external than internal (i.e. within-assemblage) filtering in shaping the trait assembly, and vice versa^21–23^. For example, if individuals need to adapt to cold temperatures at high elevations, we would expect to see lower variance of cold-adapted traits in high elevation assemblages compared to the regional pool. In contrast, a lower ratio of within-population variance to the total within-community/assemblage variance would be a signature of stronger internal filtering (e.g. interspecific competition). Specifically, we compared the intra- and interspecific variances of image features across organizational levels using *T*-statistics (‘T’ for trait)^20,21^. Since *T*-statistics require using a one-dimensional feature, we projected the 2048-dimension feature vector to a one-dimensional MDS map with precomputed cosine distance. MDS is a distance-based dimension reduction algorithm that tends to preserve the relative distances and overall structure of data points from the original space to the projected space of lower dimension, and is relatively free of distributional assumptions. Since we want to explore the inter-species feature distances of assemblages along elevation, MDS is a suitable dimension reduction tool.

**Fig. 4:**
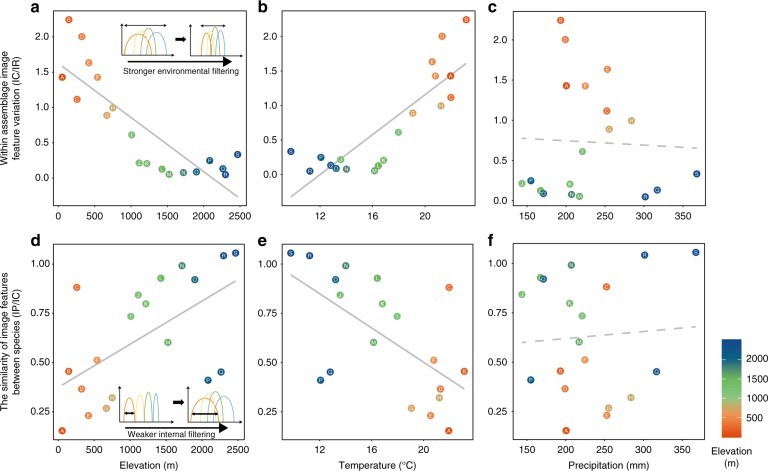
Image feature variation at the assemblage and species levels along an elevational gradient. Within-assemblage-level image feature variation (*T*_IC/IR_) in relation to **a** elevation, **b** temperature and **c** precipitation. *T*_IC/IR_ is the ratio of community/assemblage-wide variance (Individual within-community/assemblage) to total variance in the regional pool (individual within-region), assessed at the individual level. Smaller within-assemblage-level variation suggests stronger environmental filtering effect on the trait^20,21^. The similarity of image features between species at each assemblage, *T*_IP/IC_, in relation to **d** elevation, **e** temperature and **f** precipitation. *T*_IP/IC_ is the ratio of within-population variance (individual within-population) to total within-community/assemblage variance (individual within-community/assemblage). A higher *T*_IP/IC_ ratio suggests less competition among species of the same assemblage. The grey solid and dashed lines represent significant and non-significant relationships, respectively. Warmer colours correspond to lower elevations, and cooler colours to higher elevations. Source data are provided as a Source Data file

Our *T*-statistics showed that the *T*_IC/IR_, which is the ratio of community/assemblage-wide variance (individual within-community/assemblage) to total variance in the regional pool (individual within-region), decreased with increasing elevation (Fig. 4a, *F*_1,17_ = 41.03, *R*^2^ = 0.69, *p* < 0.001). This result indicates that external filtering limits image feature variation of moth assemblages at high elevations. Using the WorldClim v2 database^24^ to extract climatic data, we further found that temperature (Fig. 4b, *F*_1,17_ = 42.2, *R*^2^ = 0.70, *p* < 0.001), but not precipitation (Fig. 4c, *F*_1,17_ = 0.03, *R*^2^ = −0.06, *p* = 0.86), is the key external filter driving the pattern of lower image feature variation at high elevations. In contrast, *T*_IP/IC_, which is the ratio of within-population variance (individual within-population) to total within-community/assemblage variance (individual within-community/assemblage), decreased with decreasing elevation. This result suggests that assemblages at low elevations experience stronger internal filtering, which results in lower species overlap within an assemblage in image feature space (Fig. 4d, *F*_1,17_ = 8.6, *R*^2^ = 0.30, *p* = 0.009). Again, we found that temperature (Fig. 4e, *F*_1,17_ = 10.11, *R*^2^ = 0.34, *p* = 0.006), but not precipitation (Fig. 4f, *F*_1,17_ = 0.08, *R*^2^ = −0.05, *p* = 0.78) contributed to the lower species overlap within an assemblage in image feature space. In other words, internal filtering (interspecific competition) is stronger at low elevations with higher temperatures.

### Thermal melanism underlies elevational variation in colour diversity

To achieve a better mechanistic understanding of why image feature variation was higher at low elevations, we tested the thermal melanism hypothesis^25–28^, which predicts that darker individuals (i.e. individuals that have higher saturation and lower brightness) do better in cooler climates because they can absorb heat more quickly. We converted images from the original RGB colour space to the HSV colour space, where *H*, *S* and *V* stand for hue, saturation and value (also known as brightness), respectively. Based on a previous study showing that moths usually expose their body and forewings during rest^29^, we analysed the colour saturation and brightness of body and forewings, and then compared them to other body regions. For each species, we calculated the mean saturation (*S*) and brightness (*B*) of each species’ representative image for (i) the whole specimen (*S*_w_ and *B*_w_) and (ii) the body and forewing (*S*_f_ and *B*_f_). Furthermore, to normalize individual differences, we quantified the brightness index as the ratio of brightness of body and forewing to that of the whole specimen, *B* = *B*_f_/*B*_w_. We used the same method to calculate the saturation index, *S* = *S*_f_/*S*_w_. At the assemblage level, we calculated the average brightness and saturation indices for all species in each assemblage. We note that since hue represents categorical spectrum colours (such as red and yellow), its average cannot be calculated and, thus, is not included in the analysis.

We found that the assemblage-level brightness index decreased with increasing elevation (*F*_1,17_ = 53.6, *R*^2^ = 0.75, *p* < 0.001) and decreasing temperature (Fig. 4, *F*_1,17_ = 87.7, *R*^2^ = 0.83, *p* < 0.001). Moreover, the assemblage-level colour saturation index also significantly increased with increasing elevation (Fig. 4a, *F*_1,17_ = 7.22, *R*^2^ = 0.26, *p* = 0.016) and decreasing temperature (Fig. 4b, *F*_1,17_ = 12.76, *R*^2^ = 0.40, *p* = 0.002). These findings demonstrate that species tend to have lower brightness and higher saturation in colder environments, as predicted by the thermal melanism hypothesis^25–28^.

**Fig. 5:**
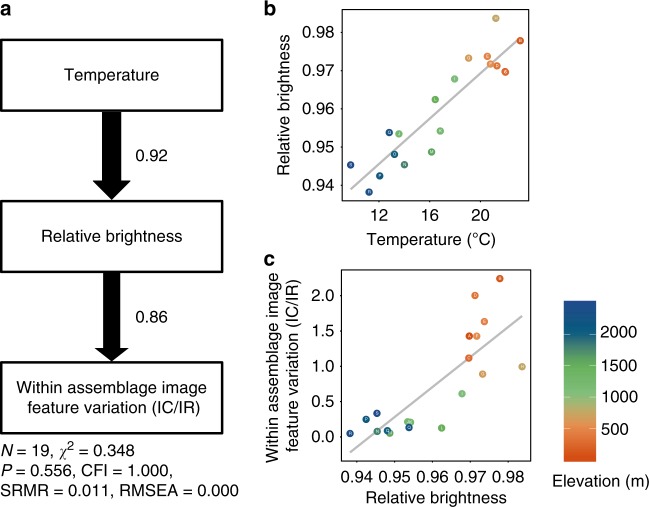
The relationships among climate, colour traits and within-assemblage image feature variation. **a** Path diagram of factors influencing within-assemblage image feature variation. The relationships between **b** mean relative brightness of species at each assemblage and temperature and **c** relative brightness and within-assemblage image feature variation (IC/IR). Path thickness is proportional to the standardized regression coefficient (shown next to the path arrows). Log transformation was applied on the IC/IR for structural equation modelling. Warmer colours correspond to lower elevations, and cooler colours to higher elevations. Source data are provided as a Source Data file

Based on our finding that cold temperatures are likely to be the key environmental factor selecting for darker colouration, we explored the potential relationship between colour darkness and colour variation in moths. Structural equation modelling, which can structure the multiple pathways to help infer the causal relationships among variables, showed that lower temperatures did indeed lead to lower relative brightness, which in turn caused lower within-assemblage image feature variation, represented by *T*_IC/IR_ (Fig. 5). Similarly, relative colour saturation also influenced the within-assemblage image feature variation such that higher colour saturation caused lower within-assemblage image feature variation, as predicted by our model (*F*_1,17_ = 6.3, *R*^2^ = 0.23, *p* = 0.02). In other words, temperature is likely to be the key environmental filtering force that generates low image feature variation in high elevation assemblages because the need for thermal regulation in colder environments constrains the colour space of moths. Indeed, our simulation analyses further confirmed that darker colour space (i.e. low brightness and high saturation in HSV colour space) leads to lower colour variation in RGB colour space (see Supplementary Note 1).

## Discussion

We have shown how deep learning provides a new approach for overcoming the difficulties often associated with quantifying complex properties of colour patterns. The high-dimensional image feature vector generated by deep learning provides an accurate and comprehensive representation of colour and shape traits and, thus, is especially suitable for studying visual phenotype diversity. Based on this breakthrough, we uncovered a surprisingly simple mechanism that colour diversity at the assemblage level is higher when environmental constraints on colour are lower in warmer environments. Many studies have demonstrated that colder environments select for darker colouration in ectotherms because dark colours can absorb heat faster^25,30^. Here we found similar patterns and show that this constraint on thermal regulation not only has pronounced effects on patterns of colouration at the assemblage level, but that it ultimately underlies broad-scale biogeographic patterns of colour variation. Previous studies have looked at colour diversity patterns at the individual level in butterflies, birds and flowers, showing that colour diversity is either not correlated with latitude or is higher at high latitudes^4,5^. However, these studies only measured relatively conspicuous colour patches in their organisms. Using deep learning, we demonstrate that patterns of subtle colour variation—as examples shown in our saliency maps^31^ (Supplementary Fig. 5), which are traits that are often difficult to define by human eyes—are likely to be more common in nature than previously realized. An obvious limitation of using the deep learning approach, however, is that we cannot know exactly which traits the feature vector encodes. Yet, by using image features to predict the elevational distribution of moths, we can study how colour diversity changes with elevation. Nevertheless, the selective pressure of this change is more difficult to determine directly, which is why we used structural equation modelling to assist in inferring how the environment shapes animal colours. It will be interesting to see if the simple rule about colour diversity we found here applies more generally to these and other organisms over broad geographic regions. Similarly, we believe that our deep learning approach—which allows AI to determine what the key traits or characters are rather than subjective human inference—can also be applied effectively to animal colouration used in other contexts, such as signalling, mimicry^1,32^ and camouflage^33^. Then, carefully interpreting the functional meanings and selective pressures of these potentially complex traits that deep learning encodes would be a fruitful and challenging step forward.

Our study further demonstrates that AI will likely facilitate the application of functional trait analysis to a wide range of ecological and evolutionary phenomena. For example, our analysis showed that simply by using functional traits, in this case moth colouration, the elevational distribution of rare species can be predicted with extremely high accuracy. Accordingly, we envision that our deep learning approach can be used to incorporate functional trait analysis with species distribution modelling^34,35^, which will be especially valuable for rare species in highly diverse taxa, particularly those experiencing changes in range size due to anthropogenic climate change. Ultimately, deep learning can help us decipher complicated natural phenomena at unpreceded depths and serve as a starting point for future exploration of the endless forms of natural morphological variation.

## Methods

### Specimen sampling and depository

Although the majority of specimens were collected through light trapping at night using a 400 W/220 V mercury lamp, a few were sampled by hand during the day or night. In total, we sampled specimens from September 2011 to September 2016 in 457 collection events at 55 localities (see map in Supplementary Fig. 10), ranging from 23 to 2470 m above sea level along an elevational gradient within a geographic range that included about 10 vegetation types of zonal forests and seven types of azonal forests^36^. All sampled specimens were deposited in Taiwan Endemic Species Research Institute, Nantou. During the 6-year collection period, we obtained 66 research permits from 23 institutions, including national parks and different forest district offices of Forestry Bureau, as well as county and city governments in Taiwan (Supplementary Note 2).

### Species identification

The identity of all specimens was confirmed by the authors with expertise in moth taxonomy. The sources for identification were based on original and subsequent references and type specimen examination, if available. For species group that are difficult or impossible to identify by appearance (e.g. the geometrid genera *Abraxas* Leach, *Lomographa* Hübner, *Maxates* Moore, the noctuid genera *Mythimna* Ochsenheime, *Callopistria* Hübner, *Ctenoplusia* Dufay, the erebid *Miltochrista* Hübner, *Simplicia* Guenée, etc.), additional morphological characters (e.g. antennal, leg, abdominal and genitalia characters), were carefully dissected for identification. Twenty-two sampled species that have not yet been formally recorded in the Taiwanese fauna or that have not yet been described as new species were identified as “sp” with additional number (1, 2, 3…) given if there is more than one species in the sampled genus. In total, we compiled a dataset including a total of 43 families, 1047 genera, 1951 species and 23,194 specimens of moths native to Taiwan. Family and genus levels follow van Nieukerken et al.^37^ and TaiCOL—Catalogue of Life in Taiwan (http://taibnet.sinica.edu.tw/home.php), respectively. The sample size distribution of elevational range size of a species is shown in Supplementary Fig. 11, and sampled species based on the number of specimen images per species in our dataset are shown in Supplementary Fig. 12.

### Image digitization and initial processing

Colour images of all (dead, spread) specimens were taken by Nikon D200/D700 with a Nikon AF Micro-Nikkor 60 mm f/2.8D/Nikon 60 mm f/2.8 G ED AF-S Micro-Nikkor (setting information: manual mode aperture as F16, speed as 1/8–1/25, ISO as 100–400/auto white balance/JPG format/ highest pixel: 10.2 and 12.1 megapixel for D200 and D700, respectively) on standardized backgrounds under the lighting of a pair of 5500 K high-frequency fluorescent luminaires. We standardized images by using the boundaries of the moth wings and body to determine the image boundaries in order to control for the potential effect of different body sizes of different species.

### Image calibration

To ensure the consistency of image quality during the acquisition of specimen images, we examined whether a sort of normalization is necessary for our colour trait analysis. We tried to equalize photography conditions among individual specimens by normalizing the brightness and saturation of a specimen by the average brightness and saturation of its background. All images were taken with the same white grid background so that we could use it for normalization. However, we found that image normalization did not significantly influence the results of trait analyses, relative to the unnormalized images, suggesting that photography conditions of all images were similar and have little impact on the results.

### Background removal

The background removal task can be essentially formulated as a kind of semantic segmentation task that aims to label every pixel in an image with a predefined category^38–40^. Recent approaches have applied CNNs to this pixel-level labelling task and achieved remarkable success in the supervised manner^40–44^. We trained a U-Net^45^ on 80% of the gold standard dataset, achieving 0.98 of mean intersection over union (mIoU) on the remaining 20% of the data. However, the U-Net’s performance degraded when applied to the TESRI dataset because of heterogeneous image backgrounds, as shown in Supplementary Fig. 13. In addition, we tried another method, Mask R-CNN, in the same setting, but the performance on our gold standard dataset was only 0.92, which was worse than an unsupervised segmentation algorithm^46^ that achieved 0.95 of mIoU. Thus, we proposed a new approach that combined an unsupervised segmentation algorithm and pseudo-labelling method to deal with the background removal task on the TESRI dataset directly. The complete process flow is summarized in Supplementary Fig. 14.

### Gold standard dataset for image segmentation

In addition to our primary dataset, we acquired another dataset, DearLep (dearlep.tw), that includes 16 families, 570 genera and 1147 species of moths in Taiwan. However, there were only 1909 available specimen images, which hindered the learning of complex colour trait patterns even though all of them have been processed in detail. Most importantly, the DearLep dataset contains the human-annotated labels of the area of the complete specimen and five dorsal parts (left and right of forewing and hindwing, respectively, and body), and we regard it as the ‘gold standard dataset’ in the following segmentation tasks.

### Unsupervised segmentation

We adopted an unsupervised segmentation algorithm that proposes a CNN to group similar pixels together with consideration of spatial continuity^46^. Regarding this background removal task, we set the number of groups at two, one for background and the other for foreground. Even though the unsupervised algorithm performs well on most images, there are still several defects, such as (i) hollow holes on wings, (ii) stains around specimen, and (iii) incompleteness due to transparent or white wings, as shown in Supplementary Fig. 15. We believe that such defects are mainly the result of shortcomings of the unsupervised algorithm, which considers RGB-based information and ignores either texture or shape information. As long as similar colours appear in both the foreground and background, defects inevitably occur. For example, if the background is white and there is a white spot on the forewings, then the spot would be recognized as background, causing a hollow hole on the output mask. To correct such defects, we retrieved the outer contours from the resulting binary mask, which works for the first two types of defect.

### Supervised segmentation based on pseudo-labels

Pseudo-labelling, which assumes those masks generated by the unsupervised method are true labels, enables us to train a background removal model in a supervised manner. Even though pseudo-labels may not be as accurate as human-annotated ones, most still provide trustworthy results. We trained a U-Net^47^ based on the pseudo-labels, since U-Net outperformed Mask R-CNN on the golden standard dataset. Learning from the pseudo-labels, U-Net successfully captured the common shape information and thus removed the background more accurately. Furthermore, this supervised model enabled us to remedy the defect of incompleteness due to transparent or white wings, as shown in Supplementary Fig. 16.

### Post-processing and manual selection

We applied a conditional random field^48^ in the post-processing step to further refine the background removal results. Lastly, we manually selected the best mask for every specimen image, resulting in a total of 23,194 background-well-removed images.

### Part model

Since most moth species expose dorsal parts of forewings and other body parts during resting, we conducted segmentation of each moth specimen image to five dorsal regions (left and right of forewing and hindwing, respectively, and body, including a pair of antenna, head, thorax and abdomen; see also ref. ^29^ for a similar approach). We used the gold standard dataset as the training dataset, with manual labelling of complete specimen images and their corresponding five parts, to segment each dorsal part out from the background-removed specimen images, as shown in Supplementary Fig. 17.

Here we implemented and compared the following three known network architectures: FC-DenseNet56^49^, DeepLabV3_plus-Res50^50^ and U-Net, all of which allowed us to segment an input image by pixel level. The comparative results are summarized in Supplementary Fig. 18, showing that U-Net achieves a higher mIoU value than the other two networks. This may be attributed to the compactness of U-Net, which avoids overfitting the model to the data and thus has better generalization performance. Ultimately, we kept 23,194 specimen images with both background removed and parts segmented.

### Problem formulation

To explore how patterns of moth colouration change with elevation, we framed the problem into a regression task that aims to predict the average elevation of every moth species. Specifically, the species average elevation prediction task took a specimen image as input *X* and outputs a real value *Y*, such that *Y* was the predicted average elevation of that species. Each species corresponded to an average elevation in the hope of establishing the relation between the colour traits of moth species and their elevational distribution. We optimized the model by the objective function of mean square error (MSE) between actual and predicted values:

1 $${\mathrm{MSE}} = \frac{1}{n}\mathop {\sum}\limits_{i = 1}^n {(Y_i - \overline {Y_i} )^2},$$

where *Y*_*i*_ is the actual average elevation of the *i*th specimen image and *Y*_*i*_ is the predicted mean elevation.

### Model architecture

Transfer learning has been shown to be successful in many computer vision tasks since common knowledge acquired from a source domain is useful to other relevant domains. Therefore, we adopt the residual network (ResNet)^18^ with 50 layers pre-trained on ImageNet as the convolutional part of our model. After the last convolutional layer of ResNet, a global average pooling layer was used to obtain a 2048-dimension feature vector. Then, a fully connected layer of 1024 neurons and a batch normalization layer were followed. Lastly, the output layer was a fully connected layer of a single neuron. Although the output layer used the linear activation function, the other layers adopt rectified linear unit as their activation function. We implemented this ResNet-based network by Keras^51^ in Python. The complete model architecture is visualized in Supplementary Fig. 1.

### Training details

All images were randomly partitioned into training (80%) and validation (20%) datasets by scikit-learn 0.20.1 module in Python 3.6.8^52^, on the condition that those images of species with only one specimen are all arranged in the training dataset. Before feeding images into our model, we resized the images to 256 by 256 pixels to unify the size of input images. Each pixel was normalized by the mean and standard deviation of images in the ImageNet dataset. During training, various data augmentation schemes—namely shifting along the *x*- and *y*-axis (±10%), scaling (±10%), rotating (±30°) and horizontal flipping—were applied independently, with each scheme having a 50% probability of occurrence, to produce additional data variety.

Our model was trained by an Adam optimizer^53^, which automatically adapts the learning rate for every parameter with the consideration of the momentum of gradients during optimization. We updated network parameters with a small initial learning rate of 5 × 10^−5^ to ensure the availability of knowledge transfer. Except for the initial learning rate, other Adam optimizer’s hyper-parameters remained as default settings in Keras. The total number of training epochs was 200, and we only retained the model at the epoch of overall minimum validation loss. We used batch normalization—which causes small deviations among different data batches that has been proven to improve the generalization of the data—to normalize the data^53^. The saliency maps were obtained by computing the gradient of outputs with respect to input images in order to highlight input regions that cause the most change in the outputs. This method enables the highlighting of salient image regions that most contribute towards the outputs.

### Statistical analyses

We assessed whether moth specimen images predicted the elevational distribution after controlling for phylogenetic effects (family and genus of species) using GLMM implemented in the R package lme4^54^. The *R*^2^squared value was calculated in reference to Nakagawa and Schielzeth^55^ with the implementation in R package MuMIn^56^. In each GLMM model, we used analysis of variance implemented in the R package car^57^ to determine whether the mixed effect had a significant effect on the predictions, and then reported the *χ*^2^ statistic and *P* value. We also assessed the intra- and interspecific variances of colour traits on individuals and assemblages using *T*-statistics implemented in the R package cati^20^. We conducted our structural equation modelling analysis in the R package Lavaan^58^.

### Climatic data

We used average monthly climatic data (i.e. only months that a given species was sampled) as a proxy for local climate for the species. The average temperature (°C) and precipitation (mm) were used to represent local climate and extracted from WorldClim v2 (30 s spatial resolution; records from 1970 to 2000^24^): http://worldclim.org/version2.

### Reporting Summary

Further information on research design is available in the Nature Research Reporting Summary linked to this article.

## Supplementary information

Supplementary Information

Reporting Summary

Source Data

## Data Availability

All original images and relavant metadata are available in the Dataset of Moth Specimens from the Taiwan Endemic Species Research Institute (TESRI) published on the GBIF website (10.15468/kjjlnf) under license CC BY 4.0. The polygons of administrative area of Taiwan used in Supplementary Fig. 10 are published on http://data.gov.tw/dataset/7442 under Open Government Data License, version 1.0. The source data underlying Figs. 1 and 3–5 and Supplementary Figs. 2–4 and 6–9 are provided as a Source Data file.

## Code availability

Fully-functional and executable codes are published on our GitHub repository (https://github.com/twcmchang/colorful-moth). We not only demonstrate how to remove heterogeneous backgrounds in specimen images, but also show how to segment each moth specimen image into five dorsal parts, including forewings, hindwings and body. The sample results are shown in the repository, and the well-trained models were uploaded for future research. See the ‘readme file’ in this repository for additional details.
