# Supplementary material for: Artificial intelligence reveals environmental constraints on colour diversity in insects
Source: Nat Commun. 2019 Oct 7;10:4554. doi: 10.1038/s41467-019-12500-2 (PMC6779759; doi:10.1038/s41467-019-12500-2)
Supplement: Supplementary file 1 — Supplementary Information [file 41467_2019_12500_MOESM1_ESM.pdf]

# Supplementary Information

# Artificial intelligence reveals environmental constraints on colour diversity in insects

Wu, Chang et al.

**Table of Contents**

Supplementary Figures 1-18_______page 1 - 18
Supplementary Note 1____________page 19
Supplementary Table 1___________page 20
Supplementary Figure 19_________page 21
Supplementary Note 2____________page 22

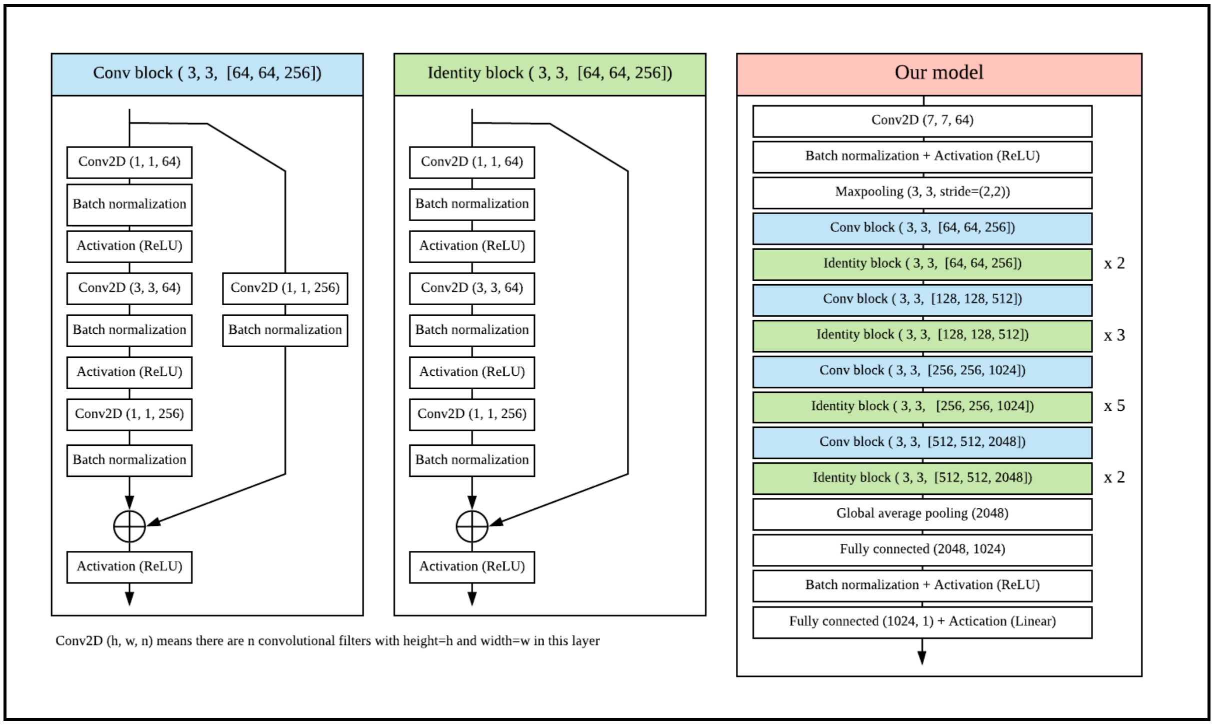


**Supplementary Figure 1**. Our model structure for average elevation prediction is on the right. Left: a "Conv block" where has a convolutional operation on the shortcut path. Middle: an "Identity block" whose shortcut path is equivalent to an identical mapping. Right: our final model.

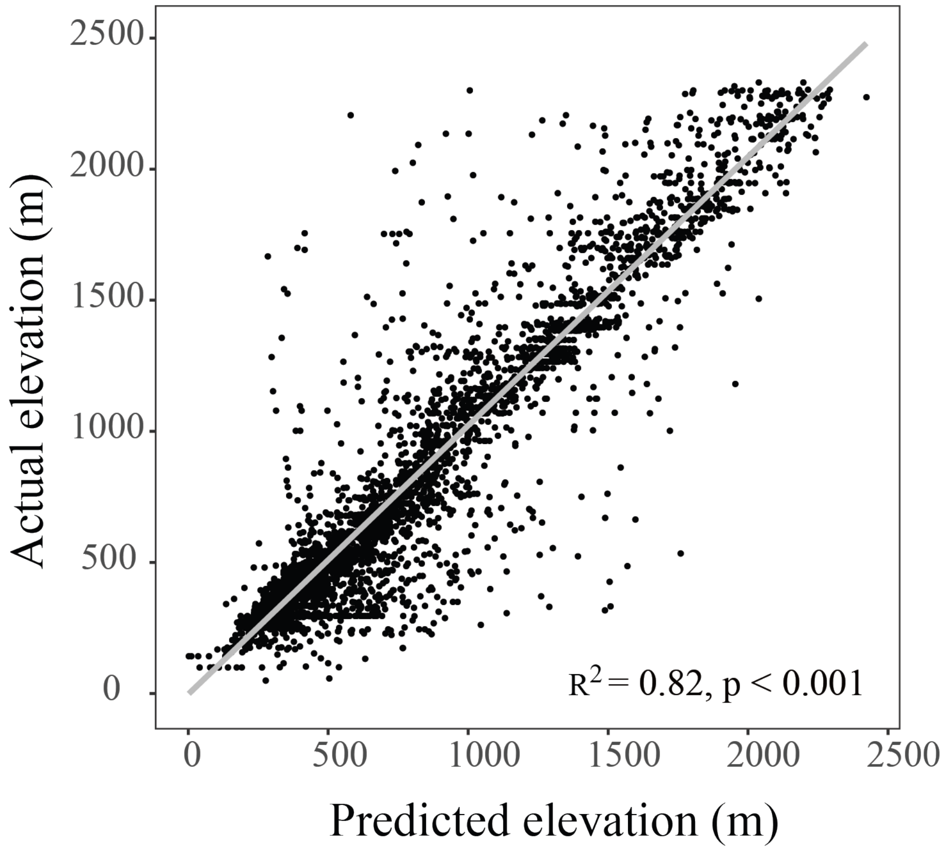


**Supplementary Figure 2.** Comparison between the predicted mean elevation of moth individuals within a species, without averaging the predicted elevations of individual images, generated by a deep learning model and the actual mean elevation of species in the validation dataset. Source data are provided as a Source Data file.

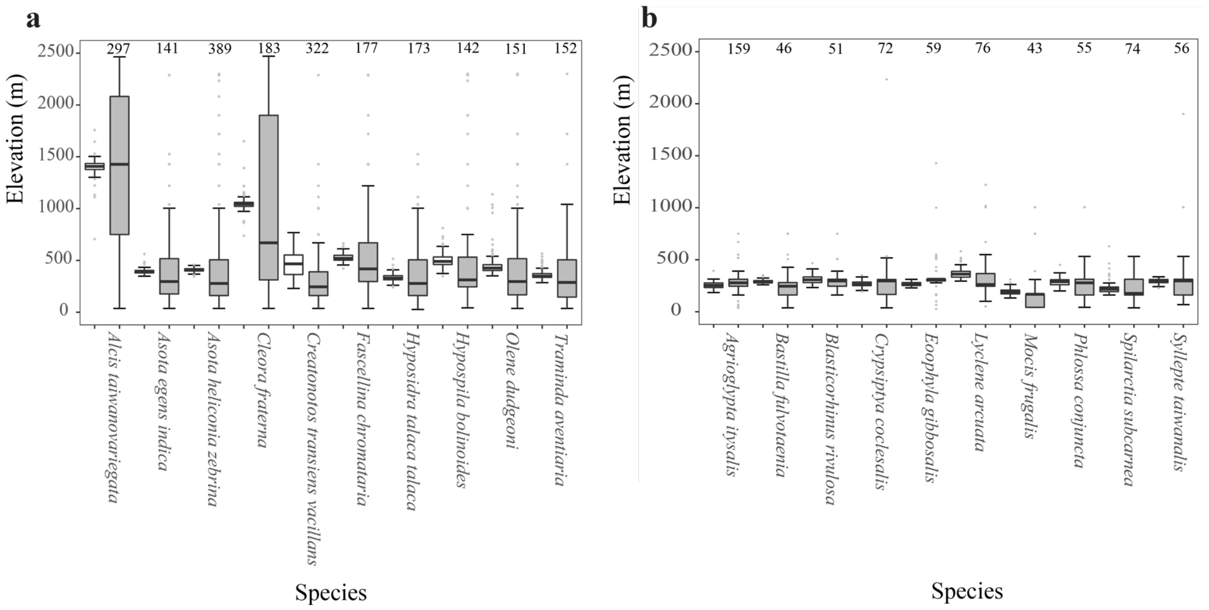


**Supplementary Figure 3.** Species selected for comparing the predicted elevational (left, white) and actual elevational ranges (right, grey). The number above the actual elevational range represents the number of sampled individuals per species. **a**. The widely distributed species (i.e., species elevational range size ≥ the median of all species elevational range sizes) with the top 10 sample sizes. **b**. the narrowly distributed species (species elevational range size < the median of all species elevational range sizes) with the top 10 sample sizes. In box-and-whiskers diagrams, boxes indicate median and upper and lower quartile and whiskers indicate range of data. Source data are provided as a Source Data file.

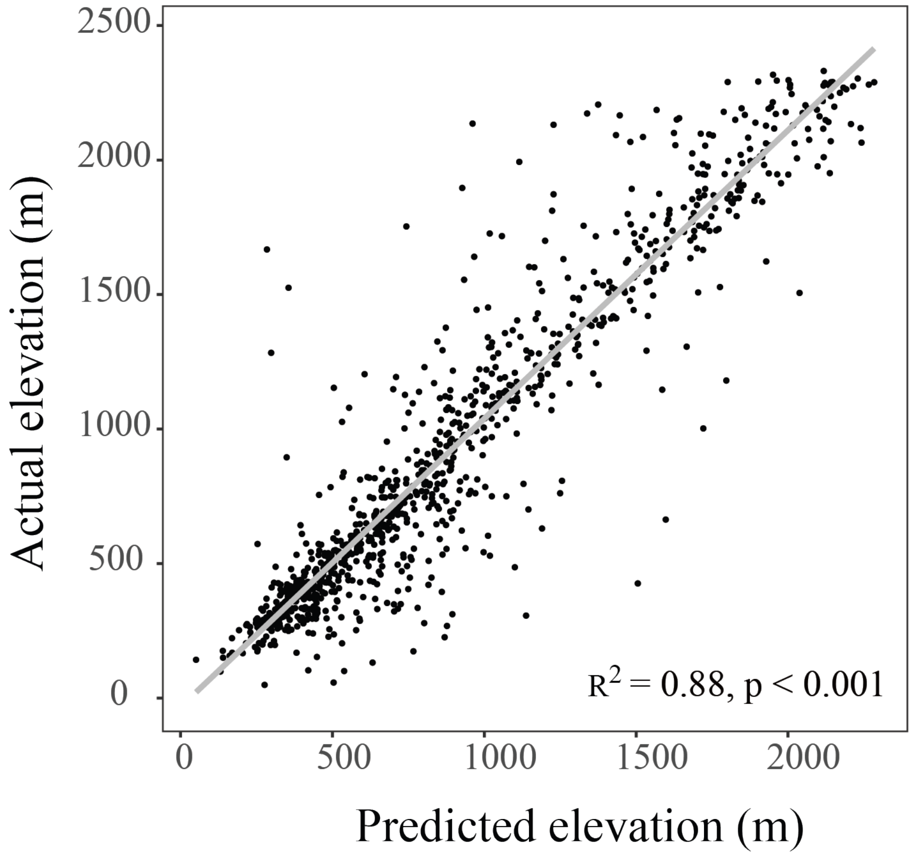


**Supplementary Figure 4.** Comparison between the predicted mean elevation of moth species, excluding species (n = 510, 26%) that only appeared in one location, generated by a deep learning model and the actual mean elevation of species in the validation dataset. Source data are provided as a Source Data file.

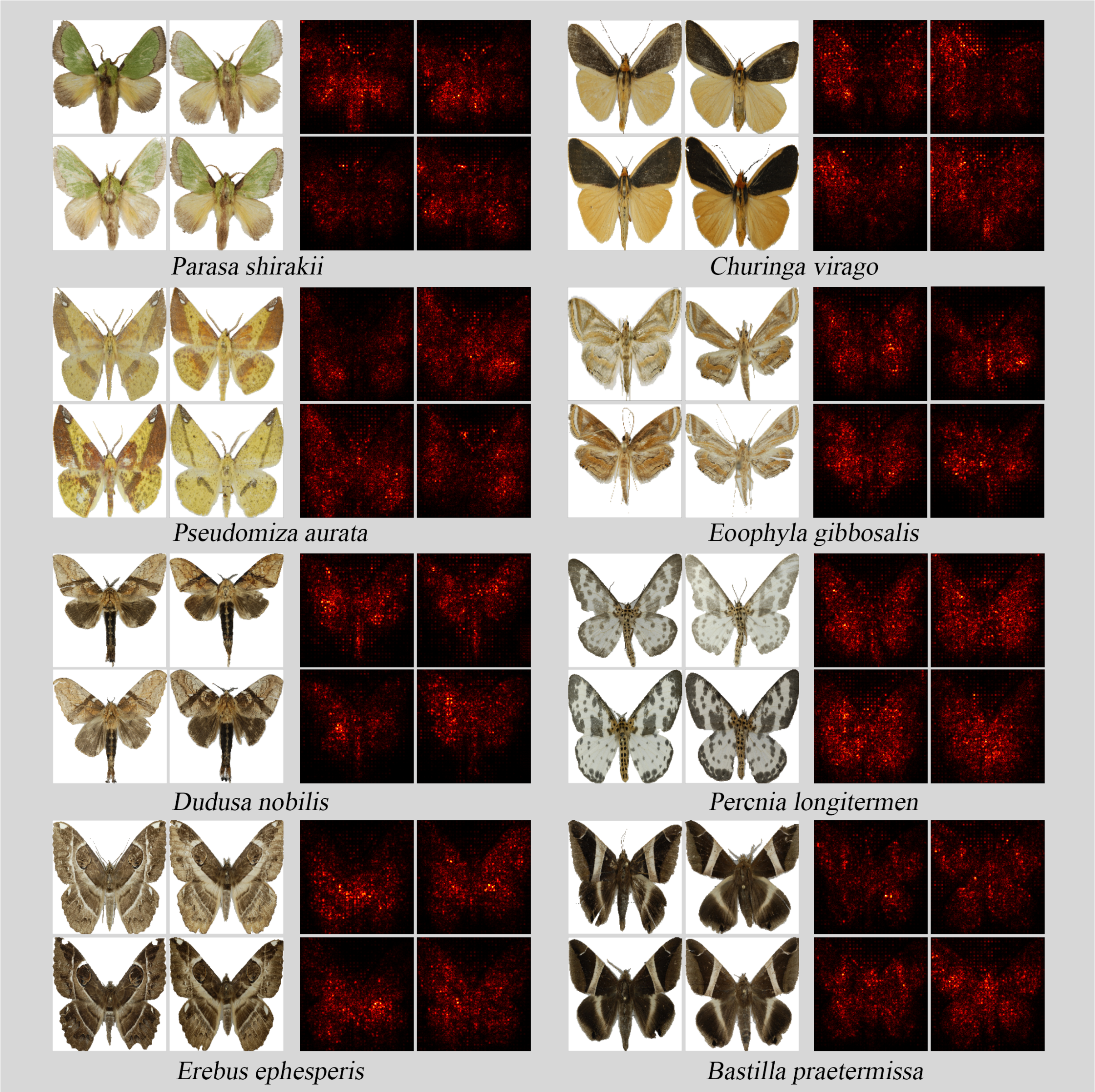


**Supplementary Figure 5**. The saliency maps and their original images of eight arbitrarily selected species. The saliency maps were obtained by computing the gradient of outputs with respect to input images in order to highlight input regions that cause the most change in the output. This method enables the highlighting of salient image regions that most contribute towards the outputs. In these figures, those pixels contributing more to the prediction of elevation are highlighted more intensely.

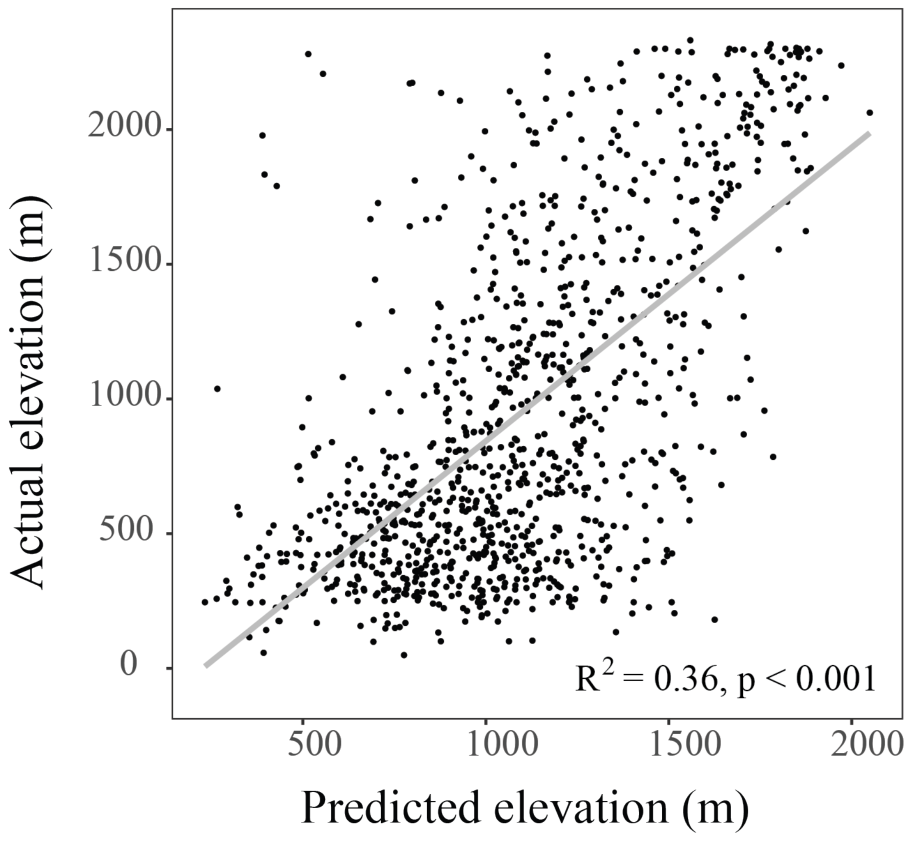


**Supplementary Figure 6.** Comparison between the predicted mean elevation of greyscale images of moth species generated by a deep learning model and the actual mean elevation of species in the validation dataset. Source data are provided as a Source Data file.

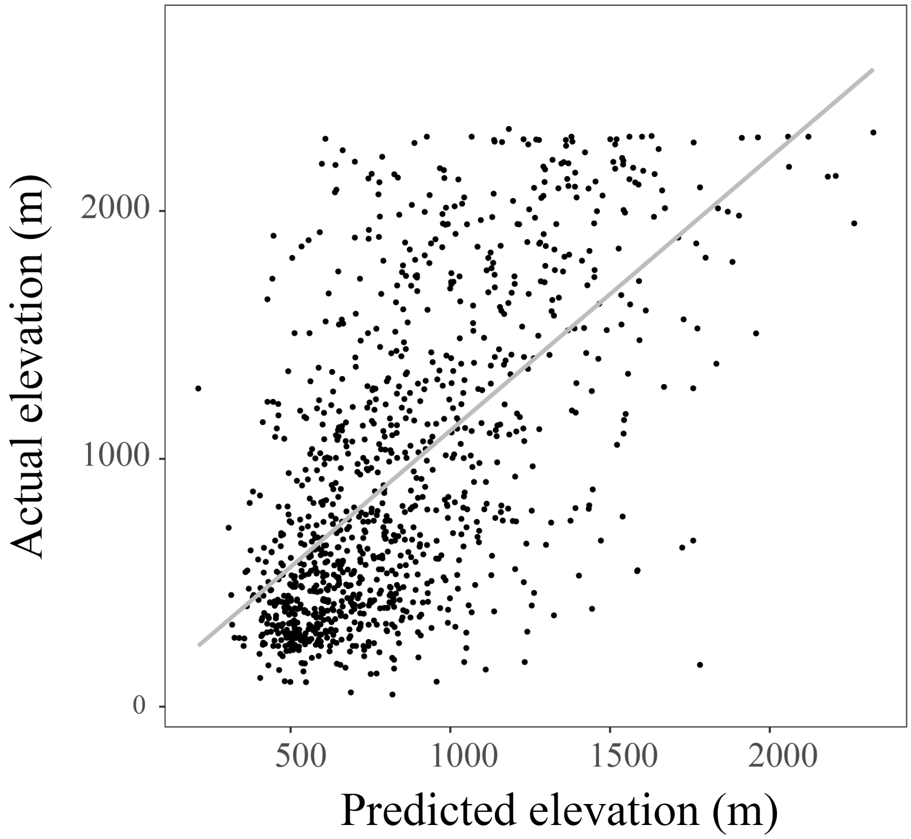


**Supplementary Figure 7.** Comparison between the predicted mean elevation of silhouette images of moth species generated by a deep learning model and the actual mean elevation of species in the validation dataset. Source data are provided as a Source Data file.

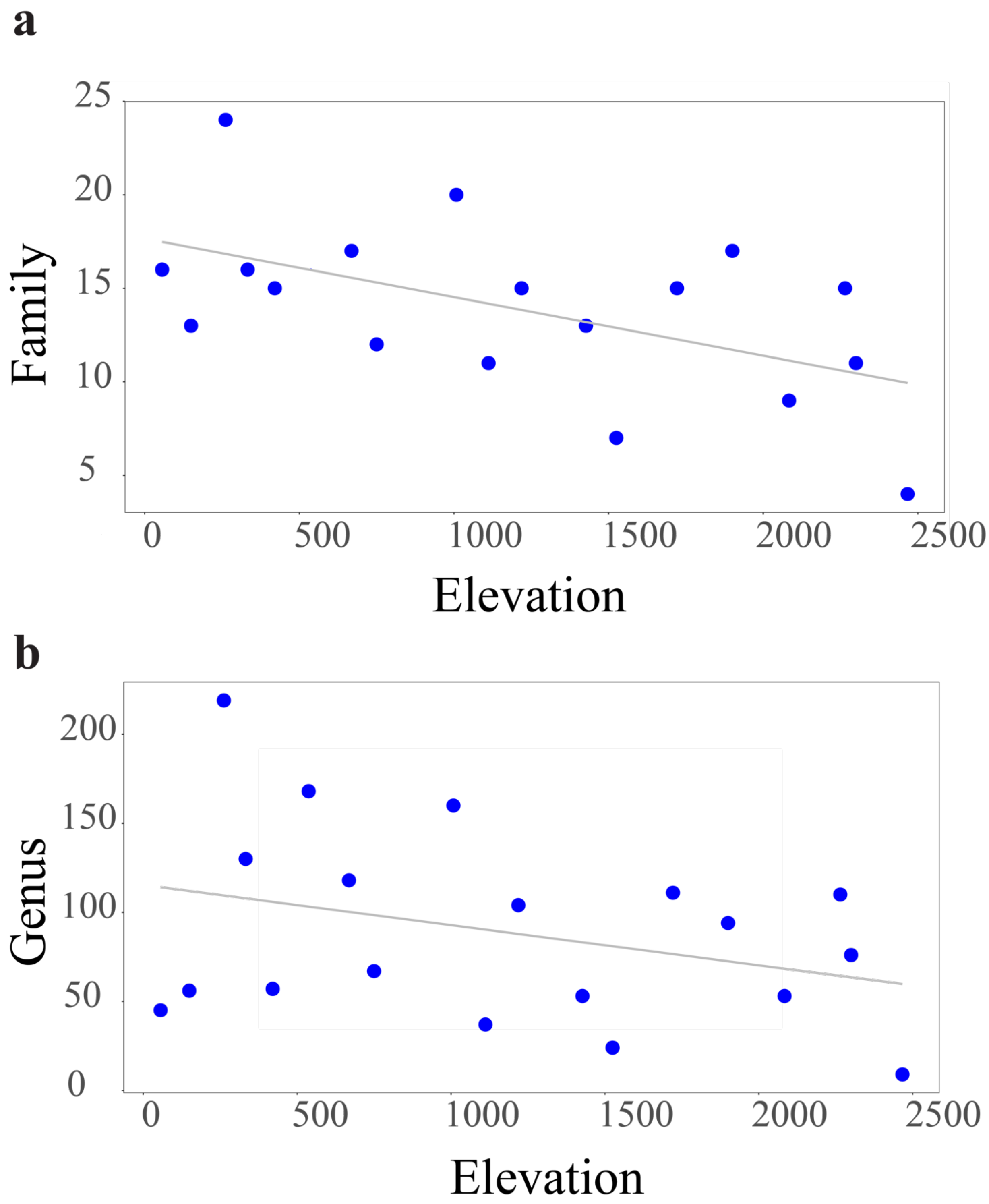


**Supplementary Figure 8.** The trend of family (a) and genus number (b) along the elevational gradient. There is a significant trend for families along elevational gradient (GLM, $R^2$=0.26, p=0.015), but not for genera (GLM, $R^2$=0.057, p=0.17). Source data are provided as a Source Data file.

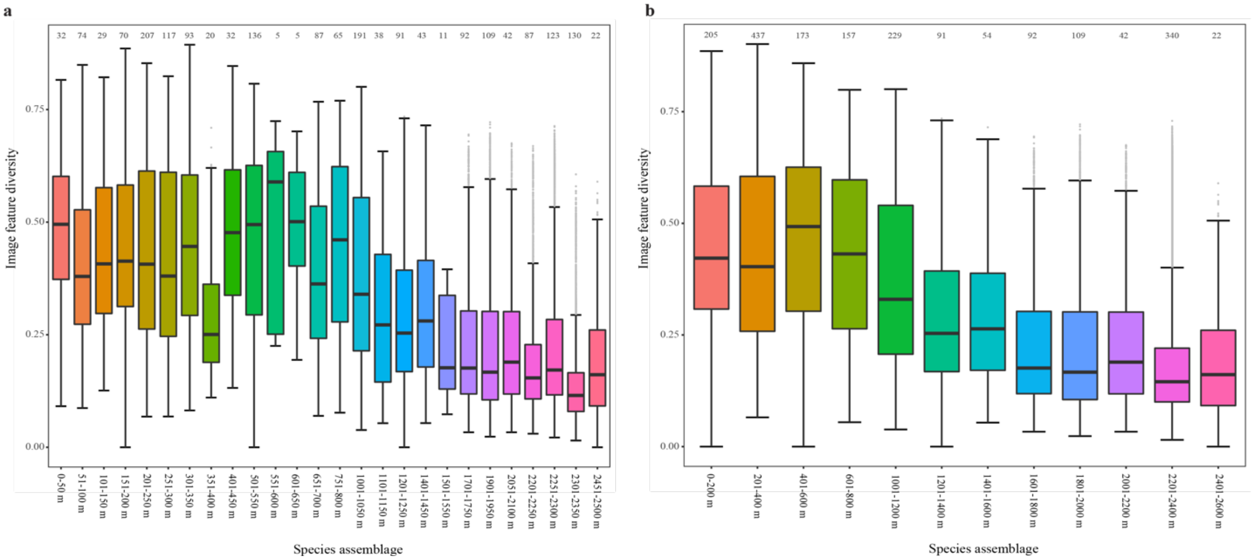


**Supplementary Figure 9.** Comparison among different assemblage divisions through different elevational intervals for analyzing within-assemblage image feature diversity of moths. a. 26 species assemblages divided by 50 m elevational intervals (started from 0 to 50 m above sea level and so on, 24 intervals have no collection event). **b**. 12 species assemblages divided by 200 m elevational intervals (started from 0 to 200 m above sea level and so on, 1 interval has no collection event). Within-assemblage image feature diversity was defined as the cosine distance of their 2048-dimension feature vectors between any two species within the same assemblage. Boxes indicate median and upper and lower quartile, and whiskers indicate range of data. Source data are provided as a Source Data file.

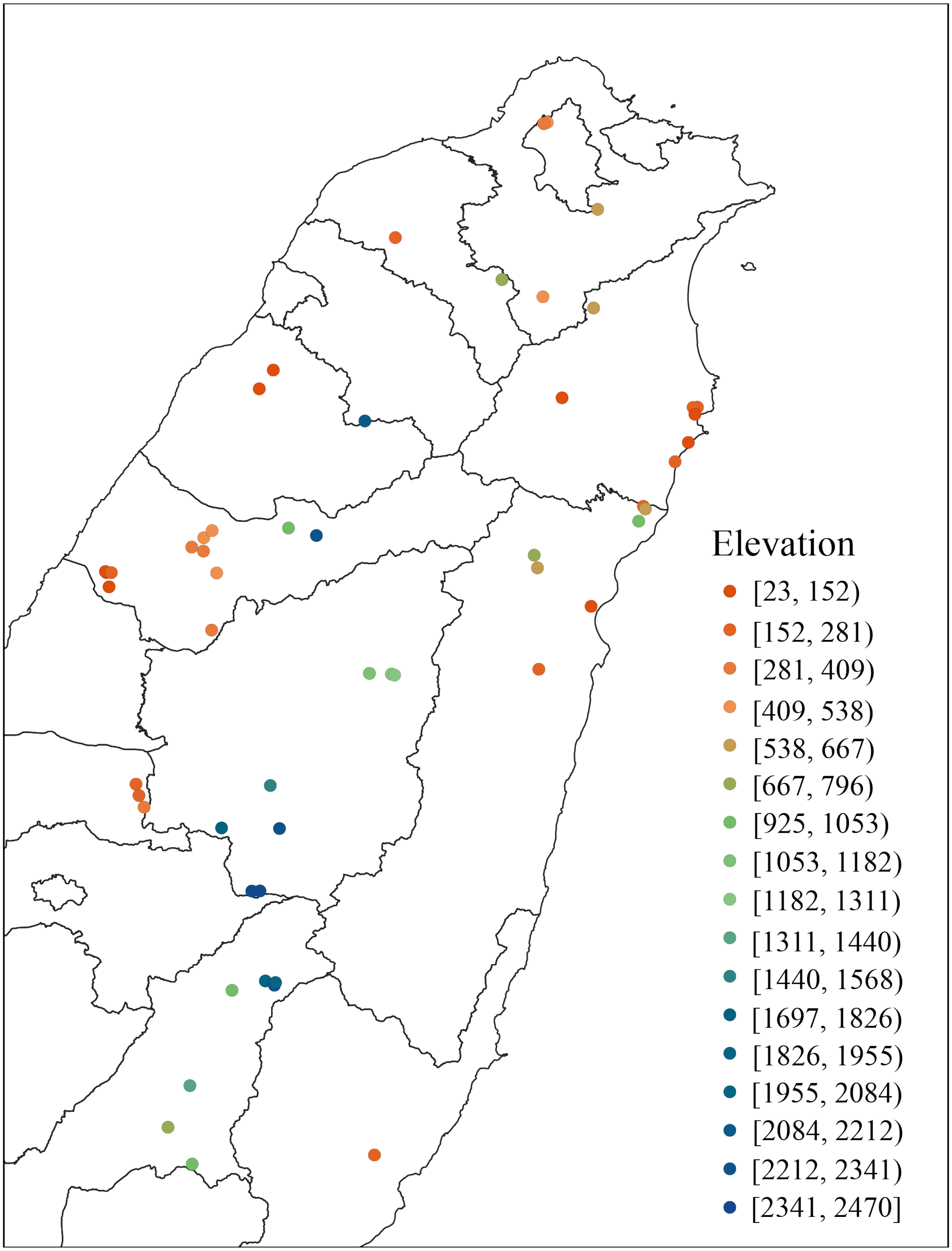


**Supplementary Figure 10.** Map of 55 moth collection localities in Taiwan. The map is generated with open-sourced QGIS 3.4 under GNU General Public License.

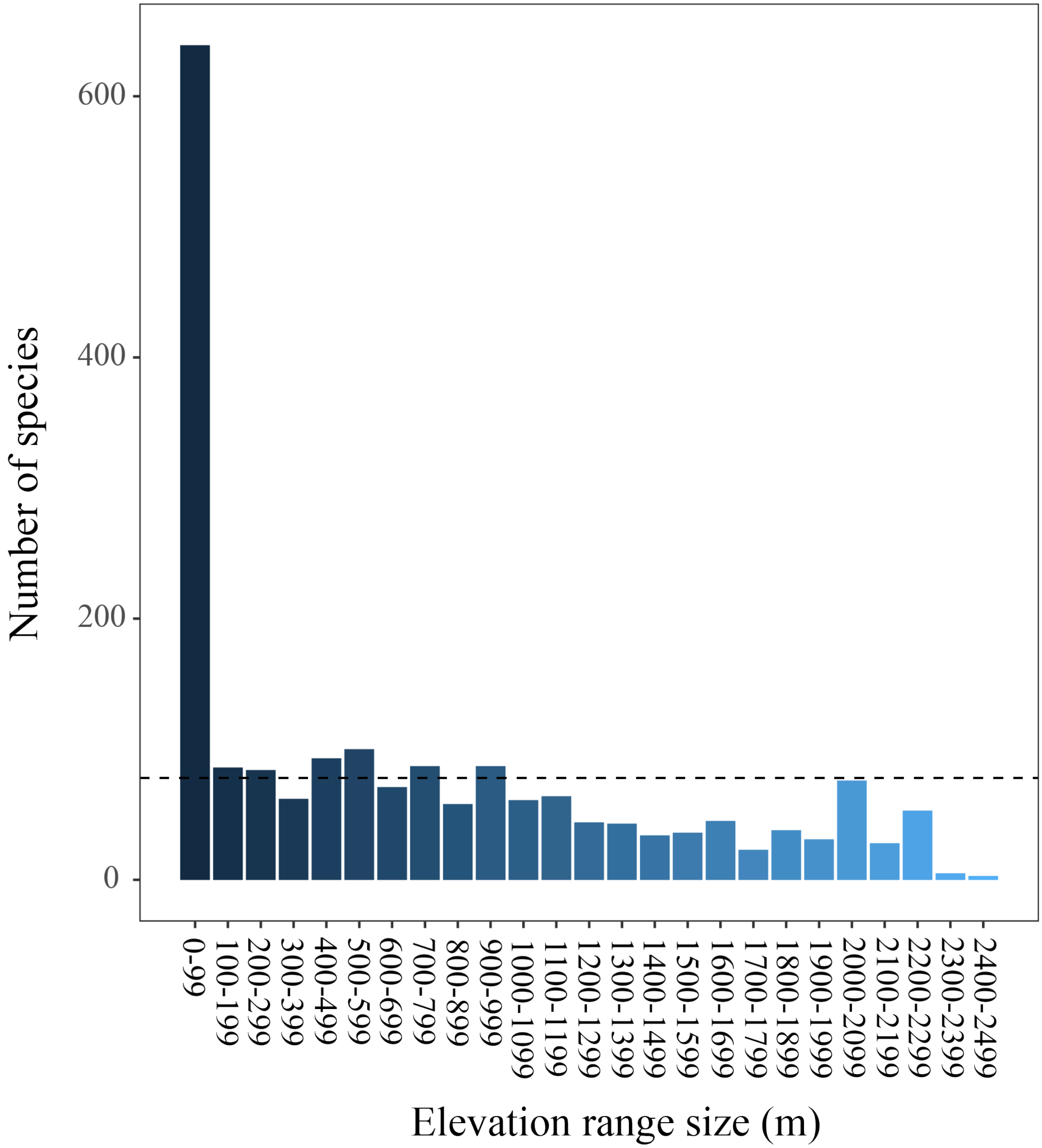


**Supplementary Figure 11.** Histogram of species numbers at different elevation range sizes.

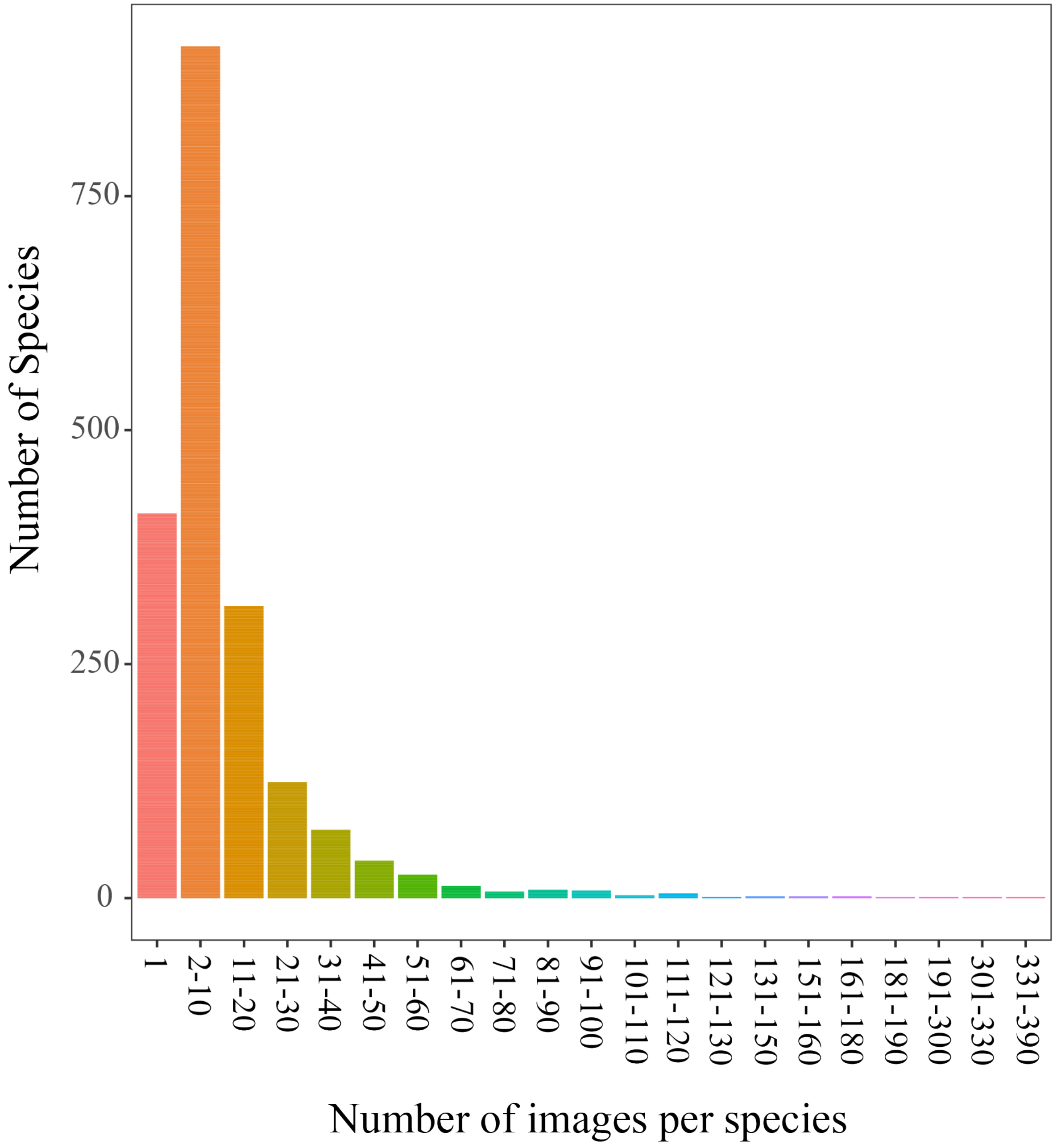


**Supplementary Figure 12.** The distribution of sampled species based on the number of images of a given species.

| Original image | U-Net mask | Unsupervised mask |
|---|---|---|
| 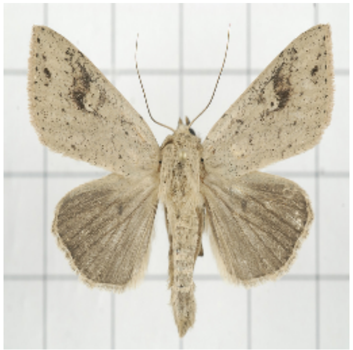 | 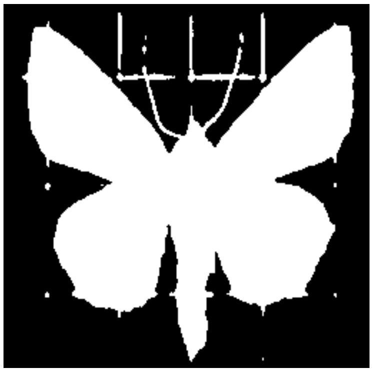 | 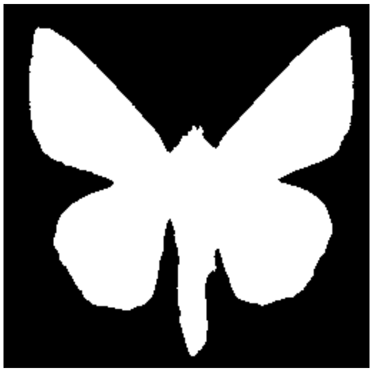 |
| 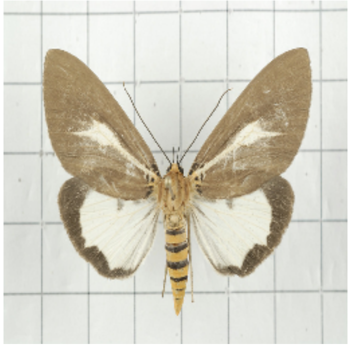 | 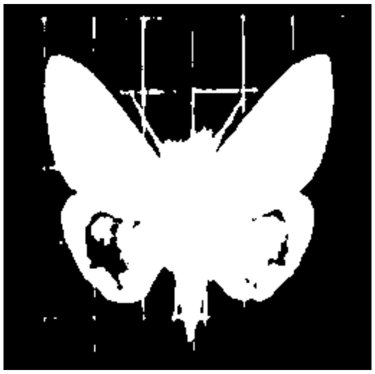 | 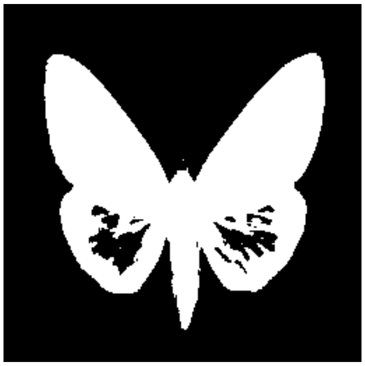 |
| 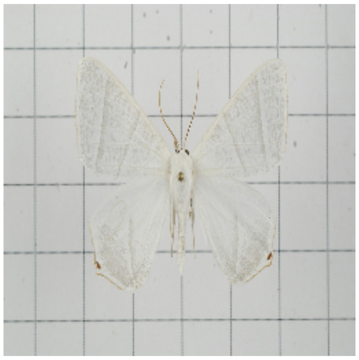 | 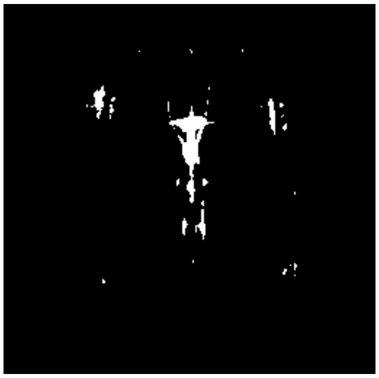 | 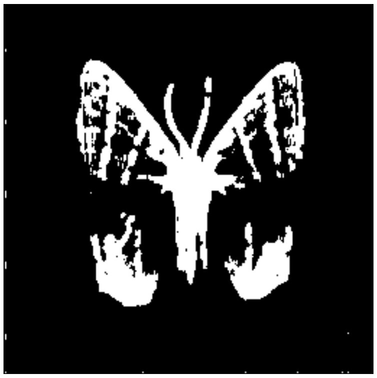 |

**Supplementary Figure 13.** Results of the U-Net model trained on the gold standard dataset and the unsupervised method, respectively. Due to the systematic difference between the gold standard dataset and the Taiwan Endemic Species Research Institute (TESRI) dataset, the supervised model trained on the gold standard dataset did not perform well as expected. Due to different image sources and variation in photographic quality between the TESRI dataset and the gold standard dataset, applying the trained supervised model to the TESRI dataset did not give as robust results as the gold standard dataset. This suggested using the TESRI dataset in a semi-supervised manner (see Methods).

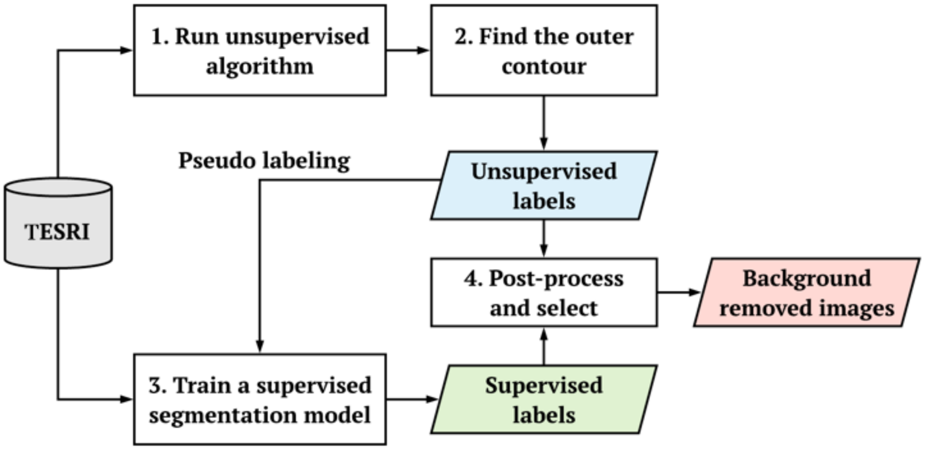


**Supplementary Figure 14.** The complete background removal process flow for the Taiwan Endemic Species Research Institute (TESRI) dataset.

| (i) hollow holes | (ii) stains around specimen | (iii) incompleteness |
|---|---|---|
| 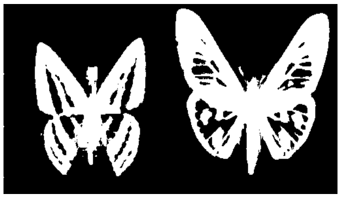 | 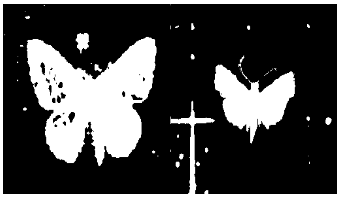 | 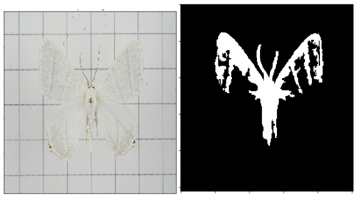 |

**Supplementary Figure 15.** Three types of defects in the unsupervised segmentation results. In (iii), the left image displays the specimen image, and the right image is its unsupervised mask.

| (a) Original image<br>(the TESRI dataset) | (b) Unsupervised mask<br>(Kanezaki, A.'s approach) | (c) Supervised mask<br>(trained on pseudo labels) |
|---|---|---|
| 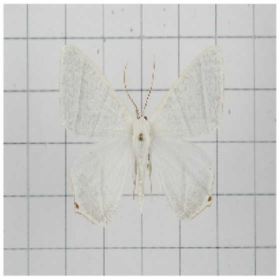 | 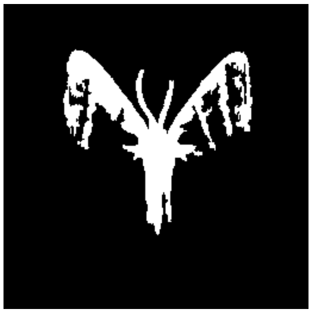 | 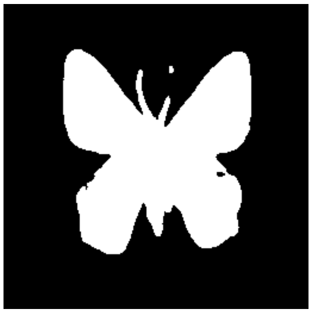 |

**Supplementary Figure 16.** Comparisons between unsupervised and supervised background removal results. The supervised model better captures shape information and enables the recovery of incompleteness defects in the unsupervised result.

| Moth | Body | Forewing (Left, Right) | | Hindwing (Left, Right) | |
|---|---|---|---|---|---|

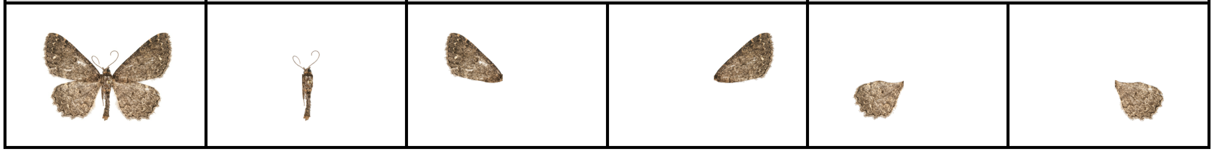

**Supplementary Figure 17.** The image on the left is the image of the moth from gold standard dataset, the others on the right are the five components respectively. (body, left-forewing, right-forewing, left-hindwing, and right-hindwing).

<table>
<tr><td>FC-DenseNet56</td><td>mean IoU = 0.9198</td></tr>
<tr><td colspan="2">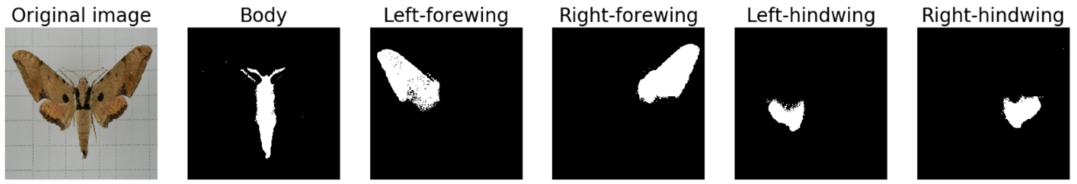
</td></tr>
<tr><td>DeepLabV3_plus-Res50</td><td>mean IoU = 0.9282</td></tr>
<tr><td colspan="2">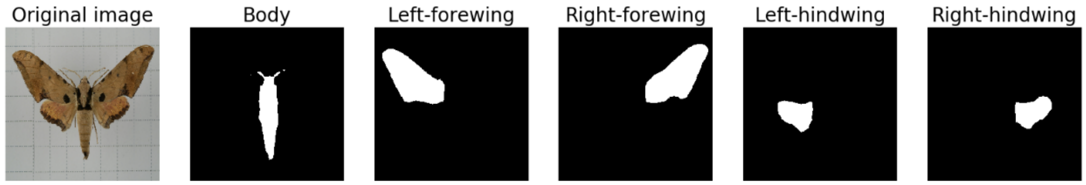
</td></tr>
<tr><td>U-Net</td><td>Mean IoU = 0.9613</td></tr>
<tr><td colspan="2">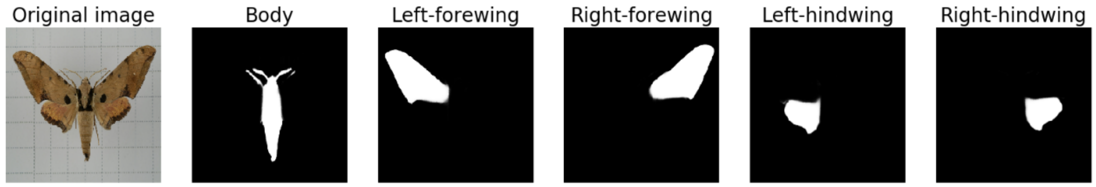
</td></tr>
</table>

**Supplementary Figure 18.** Examples of segmentation results of FC-DenseNet56, DeepLabV3_plus-Res50, and U-Net on the gold standard human-labeled dataset.

**Supplementary Note 1**

**Trade-off between colour diversity and functional colour traits**. From our structural equation modeling analysis, we found that lower relative brightness and higher relative colour saturation are related to lower image feature variation. Here we further provide a straightforward explanation to the finding by simple mathematical analysis of colour variation.

**Mathematical analysis**. We start with the definition of the HSV to RGB conversion formula and take its derivative with respect to saturation and brightness to investigate how saturation and brightness influence colour variation.

Given a HSV colour $(h, s, v)$, where $h \in [0,360)$, $s \in [0,1]$, and $v \in [0,1]$, we define that

$$h_i \equiv \left\lfloor \frac{h}{60} \right\rfloor \quad (1)$$

$$f = \frac{h}{60} - h_i \quad (2)$$

$$p = v \times (1 - s) \quad (3)$$

$$q = v \times (1 - f \times s) \quad (4)$$

$$t = v \times (1 - (1 - f) \times s) \quad (5)$$

Depending on the value of $h_i$, the corresponding RGB colour $(r, g, b)$ is

$$(r, g, b) = \begin{cases} (v, t, p), & if\ h_i = 0 \\ (q, v, p), & if\ h_i = 1 \\ (p, v, t), & if\ h_i = 2 \\ (p, q, v), & if\ h_i = 3 \\ (t, p, v), & if\ h_i = 4 \\ (v, p, q), & if\ h_i = 5 \end{cases} \quad (6)$$

First, the partial derivatives with respect to brightness, $v$:

$\frac{\partial p}{\partial v} = 1 - s$, $\frac{\partial q}{\partial v} = 1 - f \times s$, and $\frac{\partial t}{\partial v} = 1 - (1 - f) \times s$. (7)

Second, the partial derivatives with respect to saturation, $s$:

$\frac{\partial p}{\partial s} = -v$, $\frac{\partial q}{\partial s} = -v \times f$, and $\frac{\partial t}{\partial s} = -(1 - f) \times v$. (8)

In the following, we assume the value of hue $(h)$ at 0, therefore $(r, g, b) = (v, t, p)$. If a small change in saturation $(\Delta s)$ occurs, the resulting changes in $(r, g, b)$ can be approximated by the Newton's method using the first order derivative, such as

$(\Delta r, \Delta g, \Delta b) = (\Delta v, \Delta t, \Delta p) \approx (\Delta v, -(1 - f) \times v \times \Delta s, -v \times \Delta s)$, (9)

showing that

$|\Delta g| \propto v$ and $|\Delta b| \propto v$. (10)

Similarly, the small change in brightness $(\Delta v)$brings about a change in $(r, g, b)$as

$(\Delta r, \Delta g, \Delta b) \approx (\Delta v, (1 - (1 - f) \times s) \times \Delta v, (1 - s) \times \Delta v)$,

revealing that

$|\Delta g| \propto (1 - s)$ and $|\Delta b| \propto (1 - s)$. (11)

These two findings, (10) and (11), disclose the underlying relationships among saturation, brightness, and the values of RGB channels, implying that both high saturation and low brightness indeed constrain the colour variation presenting in the RGB channels.

**Simulation test**. We simulated the case that two groups of colours with unequal means but equal variances of colour distribution in the HSV colour space. One colour group is designed to present the characteristics of the species assemblages at high elevations, having greater mean saturation but smaller mean brightness, therefore called 'high elevation' group; in contrast, another one stands for 'low elevation'. The detailed settings are summarized in Supplementary Table 1, where the hue value is set at 0° (red), 120° (green), or 240°(blue). As shown in Supplementary Figure 19, the simulation results agree with the previous mathematical analysis: the low elevation group has a wider range of variation in both G and B channels than the high elevation one, as expected.

**Supplementary Table 1.** The setting of two groups of colours. One represents the circumstance of high saturation and low brightness at high elevations; the other stands for the characteristics at low elevations.

| Group | H | S (distribution) | V (distribution) |
|---|---|---|---|
| High elevation | {0°, 120°, 240°} | Uniform, (0.6, 0.8] | Uniform, (0.2, 0.4] |
| Low elevation | {0°, 120°, 240°} | Uniform, (0.2, 0.4] | Uniform, (0.6, 0.8] |

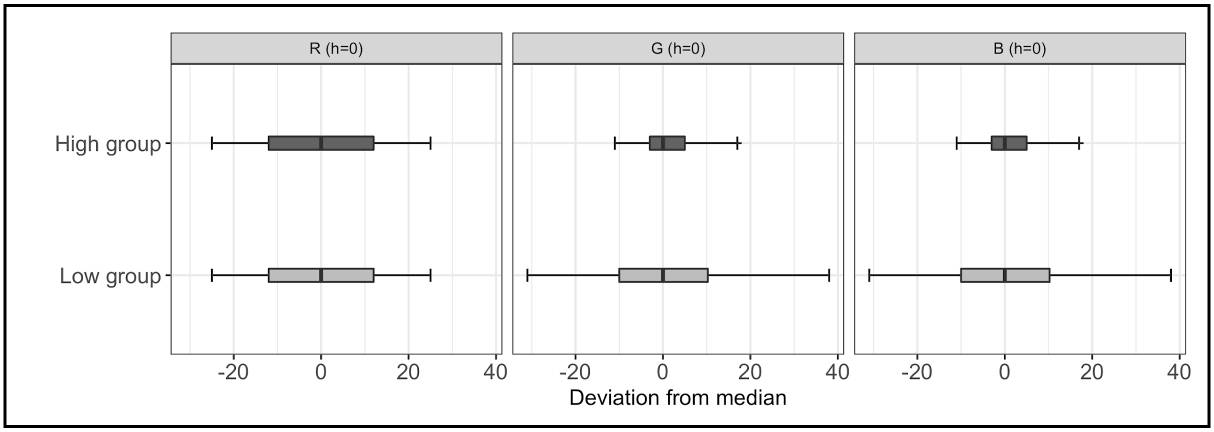


**Supplementary Figure 19.** Comparison of the statistics of deviation (from median) in R, G, B channels between high and low elevation groups. Because the deviation of both groups in the R channel is equal to the variance of brightness, there is no difference in the R channel.

**Supplementary Note 2**

Lists of collection permissions during 2011-2016 (arranged in alphabetical order)

| **Issuing authority** | **Permit number (year of issue)** |
|---|---|
| Chiayi Forest District Office | 1005103018 (2011) |
| Dongshih Forest District Office | 1023100278 & 1023102168 (2013), 1043160060 (2015), 1043109598 (2016) |
| Hsinchu City Government | 1023226309 (2013), 1043090191 (2015), 1053060185 (2016) |
| Hsinchu Forest District Office | 1002240862 (2011), 1022100359 & 1022103307 (2013), 1022114444, 1022114445 (2014), 1042100065 (2015) |
| Hualien Forest District Office | 1048100082 (2015) |
| Kaohsiung City Government | 10201950900 (2013), 10207213400 (2014), 10400005500 (2015), 10407283700 (2016) |
| Lukuei Reseach Center | 1042100001 (2015) & 1042264990 (2016) |
| Luodong Forest District Office | 1001100996 (2011), 1041100035 (2015), 1041104407 (2016) |
| Miaoli County Government | 1020060949 (2013), 1020261167 (2014), 1040000200 (2015), 1050004100 (2016) |
| Nantou County Government | 1020087884 & 1024100458 (2013), 1020257492 (2014), 1040003835 (2015), 1040263167 (2016) |
| Nantou Forest District Office | 1024240361 (2013), 1024112670 & 1024112671 (2014) |
| New Taipei City Government | 1023246940 (2014) |
| Taichung City Government | 1020054729 & 1020054731 (2013), 1020250016 (2014), 1040001000 (2015), 1050000051 (2016) |
| Taichung County Government | 990403753 (2011) |
| Taijian National Park | 1050000079 (2016) |
| Taitung County Government | 1020057921 (2013), 1020248252 (2014) |
| Taitung Forest District Office | 1007101317 (2011), 1037240466 (2014), 1047100033 (2015), 1047107928 (2016) |
| Taoyuan County Government | 1020042127 (2013) |
| Taroko National Park Headquarters | 990014698 & 990014699 (2011), 1020014578 (2014), 1040000136 (2015) |
| Yangminshan National Park | 2013252 (2013), 20131503 (2014), 20151242 (2015), 20150099 (2016) |
| Yilan County Government | 1040216585 (2016) |
| Yunlin County Government | 1020048685 (2013), 1025527638 (2014) |
| Yushan National Park Headquarters | 1030001509 (2014), 1040000053, 1040002293 (2015), 1040004994 (2016) |
