# Supplementary material for: Artificial intelligence reveals environmental constraints on colour diversity in insects
Source: Nat Commun. 2019 Oct 7;10:4554. doi: 10.1038/s41467-019-12500-2 (PMC6779759; doi:10.1038/s41467-019-12500-2)
Supplement: Supplementary file 2 — Reporting Summary [file 41467_2019_12500_MOESM2_ESM.pdf]

nature research

Corresponding author(s): Sheng-Feng Shen and Sheng-Wei Chen

Last updated by author(s): Aug 23, 2019

Reporting Summary

Nature Research wishes to improve the reproducibility of the work that we publish. This form provides structure for consistency and transparency in reporting. For further information on Nature Research policies, see Authors & Referees and the Editorial Policy Checklist .

Statistics

For all statistical analyses, confirm that the following items are present in the figure legend, table legend, main text, or Methods section.

n/a	Confirmed	
☐	☒	The exact sample size (n) for each experimental group/condition, given as a discrete number and unit of measurement
☐	☒	A statement on whether measurements were taken from distinct samples or whether the same sample was measured repeatedly
☐	☒	The statistical test(s) used AND whether they are one- or two-sided<br>*Only common tests should be described solely by name; describe more complex techniques in the Methods section.*
☐	☒	A description of all covariates tested
☐	☒	A description of any assumptions or corrections, such as tests of normality and adjustment for multiple comparisons
☐	☒	A full description of the statistical parameters including central tendency (e.g. means) or other basic estimates (e.g. regression coefficient) AND variation (e.g. standard deviation) or associated estimates of uncertainty (e.g. confidence intervals)
☐	☒	For null hypothesis testing, the test statistic (e.g. F, t, r) with confidence intervals, effect sizes, degrees of freedom and P value noted<br>*Give P values as exact values whenever suitable.*
☒	☐	For Bayesian analysis, information on the choice of priors and Markov chain Monte Carlo settings
☒	☐	For hierarchical and complex designs, identification of the appropriate level for tests and full reporting of outcomes
☐	☒	Estimates of effect sizes (e.g. Cohen's d, Pearson's r), indicating how they were calculated

Our web collection on statistics for biologists contains articles on many of the points above.

Software and code

Policy information about availability of computer code

Data collection	No software was used.
Data analysis	We used R for statistical analyses and python to construct the deep learning models.

For manuscripts utilizing custom algorithms or software that are central to the research but not yet described in published literature, software must be made available to editors/reviewers. We strongly encourage code deposition in a community repository (e.g. GitHub). See the Nature Research guidelines for submitting code & software for further information.

Data

Policy information about availability of data

All manuscripts must include a data availability statement. This statement should provide the following information, where applicable:

- Accession codes, unique identifiers, or web links for publicly available datasets
- A list of figures that have associated raw data
- A description of any restrictions on data availability

All original images and relavant metadata are available in the Dataset of Moth Specimens from the Taiwan Endemic Species Research Institute (TESRI) published on the GBIF website (https://doi.org/10.15468/kjjlnf) under license CC BY 4.0. The source data underlying Figs. 1 and 3–5 and Supplementary Figs. 2-4 and 6-9 are provided as a Source Data file. The polygons of administrative area of Taiwan used in Supplementary Fig. 10 are published on http://data.gov.tw/dataset/7442 under Open Government Data License, version 1.0.

Field-specific reporting

Please select the one below that is the best fit for your research. If you are not sure, read the appropriate sections before making your selection.

Ecological, evolutionary & environmental sciences study design

All studies must disclose on these points even when the disclosure is negative.

Study description	We use deep learning to analyse associations among elevation, climate and phenotype across ca. 2000 moth species in Taiwan.
Research sample	We totally sampled 43 families, 1,047 genera, 1,951 species, 23,194 specimens of moths native to Taiwan.
Sampling strategy	We collected as many samples as possible for a species during the sampling period. An extremely large sample size was collected. In total, we sampled specimens from September 2011 to September 2016 in 457 collection events at 55 localities, ranging from 23 to 2,470 m above sea level (a.s.l.) along an elevational gradient within a geographic range that included about 10 vegetation types of zonal forests and seven types of azonal forests. In total, we compiled a dataset including a total of 43 families, 1,047 genera, 1,951 species, 23,194 specimens of moths native to Taiwan.
Data collection	All sampled moth specimens were largely taken by local citizens through light trap. Image data were taken by Hsu-Hong Lin and members of Mr. Lin's research group.
Timing and spatial scale	All sampled moth specimens were taken in 55 localities once a month during 2011 and 2016.
Data exclusions	Specimen images were excluded if the background cannot be removed.
Reproducibility	All the results and figures can be reproduced by the data provided.
Randomization	All images were randomly partitioned into training (80%) and validation (20%) datasets by scikit-learn 0.20.1 module in Python 3.6.8.
Blinding	Blinding is not relevant to our study.

Did the study involve field work? ☒ Yes ☐ No

Field work, collection and transport

Field conditions	Moth samples were mostly collected near natural forest edges in the evening in all weather conditions.
Location	we sampled specimens from September 2011 to September 2016 in 457 collection events at 55 localities (see map in Supplementary Fig. 10), ranging from 23 to 2,470 m above sea level (a.s.l.) along an elevational gradient within a geographic range that included about 10 vegetation types of zonal forests and seven types of azonal forests.
Access and import/export	Samples were collected in the habitats can be accessed by car. Detailed information regarding research permits are available in Supplementary Note 2.
Disturbance	We collect samples only once a month at the same location to minimized the disturbance frequency and impacted regions.

Reporting for specific materials, systems and methods

We require information from authors about some types of materials, experimental systems and methods used in many studies. Here, indicate whether each material, system or method listed is relevant to your study. If you are not sure if a list item applies to your research, read the appropriate section before selecting a response.

Materials & experimental systems

n/a	Involved in the study
☒	☐ Antibodies
☒	☐ Eukaryotic cell lines
☒	☐ Palaeontology
☐	☒ Animals and other organisms
☒	☐ Human research participants
☒	☐ Clinical data

Methods

n/a	Involved in the study
☒	☐ ChIP-seq
☒	☐ Flow cytometry
☒	☐ MRI-based neuroimaging

Animals and other organisms

Policy information about studies involving animals; ARRIVE guidelines recommended for reporting animal research

Laboratory animals	N/A

Wild animals	Most moth specimens were attracted by mercury lamps at night and killed with ammonia or Ethyl Acetate onsite ingeniously. Then, the samples were taken back to the laboratory and made into specimens.
Field-collected samples	N/A
Ethics oversight	No formal ethical approval was required except sampling permit because agencies in Taiwan (and many other places) only review vertebrates studies.

Note that full information on the approval of the study protocol must also be provided in the manuscript.
